# Effect of Acupoint Hot Compress on Postpartum Urinary Retention After Vaginal Delivery: A Randomized Clinical Trial

**DOI:** 10.1001/jamanetworkopen.2022.13261

**Published:** 2022-05-23

**Authors:** Yuhang Zhu, Fangfang Wang, Jue Zhou, Shuiqin Gu, Lianqing Gong, Yaoyao Lin, Xiaoli Hu, Wei Wang, Aihua Zhang, Dongmei Ma, Chunxiao Hu, Yan Wu, Lanzhong Guo, Limin Chen, Leiyin Cen, Yan He, Yuqing Cai, Enli Wang, Honglou Chen, Jing Jin, Jinhe Huang, Meiyuan Jin, Xiujuan Sun, Xiaojiao Ye, Linping Jiang, Ying Zhang, Jian Zhang, Junfei Lin, Chunping Zhang, Guofang Shen, Wei Jiang, Liuyan Zhong, Yuefang Zhou, Ruoya Wu, Shiqing Lu, Linlin Feng, Hong Guo, Shanhu Lin, Qiaosu Chen, Jinfang Kong, Xuan Yang, Mengling Tang, Chang Liu, Fang Wang, Xiao-Yang Mio Hu, Hye Won Lee, Xinfen Xu, Rong Zhang, Nicola Robinson, Myeong Soo Lee, Jisheng Han, Fan Qu

**Affiliations:** 1 Department of Chinese Integrative Medicine, Women’s Hospital, School of Medicine, Zhejiang University, Hangzhou, China; 2 School of Food Science and Biotechnology, Zhejiang Gongshang University, Hangzhou, China; 3 Department of Obstetrics, Jiaxing Maternity and Child Health Care Hospital, Jiaxing, China; 4 Department of Obstetrics, Yiwu Maternity and Child Health Care Hospital, Yiwu, China; 5 Department of Epidemiology and Biostatistics at School of Public Health and the Fourth Affiliated Hospital, School of Medicine, Zhejiang University, Hangzhou, China; 6 Department of Obstetrics, Tongde Hospital of Zhejiang Province, Hangzhou, China; 7 Department of Obstetrics, Xianju People’s Hospital, Xianju, China; 8 Department of Obstetrics, The Women and Children Hospital of Dongyang, Dongyang, China; 9 Department of Obstetrics, Shaoxing Maternity and Child Health Care Hospital, Shaoxing, China; 10 Department of Obstetrics, Cixi Maternity and Child Health Care Hospital, Cixi, China; 11 Department of Obstetrics, Zhoushan Women and Children Hospital, Zhoushan, China; 12 Department of Obstetrics, Ruian People’s Hospital, Ruian, China; 13 Department of Obstetrics, Wenling Maternity and Child Health Care Hospital, Wenling, China; 14 Department of Obstetrics, Zhejiang Xiaoshan Hospital, Hangzhou, China; 15 Department of Obstetrics, The Second Clinical Medical College of Zhejiang Chinese Medical University, Hangzhou, China; 16 Primary Care, Population Sciences and Medical Education, Faculty of Medicine, University of Southampton, Southampton, United Kingdom; 17 Clinical Research Division, Korea Institute of Oriental Medicine, Daejeon, Republic of Korea; 18 Neuroscience Research Institute, Peking University, Beijing, China; 19 School of Health and Social Care, London South Bank University, London, United Kingdom

## Abstract

**Importance:**

Acupoint hot compress during the early postpartum period may benefit patients after a vaginal delivery, but the evidence of this effect is limited.

**Objective:**

To assess whether acupoint hot compress involving the abdominal, lumbosacral, and plantar regions could reduce the incidence of postpartum urinary retention, relieve postpartum uterine contraction pain, prevent emotional disorders, and promote lactation.

**Design, Setting, and Participants:**

This multicenter randomized clinical trial was conducted at 12 hospitals in China. Pregnant patients were screened for eligibility (n = 13 949) and enrolled after vaginal delivery (n = 1200) between January 17 and August 15, 2021; data collection was completed on August 18, 2021. After vaginal delivery, these participants were randomized 1:1 to either the intervention group or control group. Statistical analysis was based on per-protocol population.

**Interventions:**

Participants in the control group received routine postpartum care. Participants in the intervention group received routine postpartum care plus 3 sessions of a 4-hour acupoint hot compress involving the abdominal, lumbosacral, and plantar regions within 30 minutes, 24 hours, and 48 hours after delivery.

**Main Outcomes and Measures:**

The primary outcome was the incidence of postpartum urinary retention, defined as the first urination occurring more than 6.5 hours after delivery and/or use of an indwelling catheter within 72 hours after delivery. The secondary outcomes were postpartum uterine contraction pain intensity (assessed with the visual analog scale [VAS]), depressive symptoms (assessed with the Edinburgh Postnatal Depression Scale), and lactation conditions (including lactation initiation time, breastfeeding milk volume, feeding mood and times, and newborn weight).

**Results:**

Of the 1200 participants randomized, 1085 completed the study (537 in the intervention group and 548 in the control group, with a median [IQR] age of 26.0 [24.0-29.0] years). Participants in the intervention group compared with the control group had significantly decreased incidence of postpartum urinary retention (relative risk [RR], 0.58; 95% CI, 0.35-0.98; *P* = .03); improved postpartum uterine contraction pain when measured at 6.5 hours (median [IQR] VAS score, 1 [1-2] vs 2 [1-2]; *P* < .001), 28.5 hours (median [IQR] VAS score, 1 [0-1] vs 1 [1-2]; *P* < .001), 52.5 hours (median [IQR] VAS score, 1 [0-1] vs 1 [0-1]; *P* < .001), and 76.5 hours (median [IQR] VAS score, 0 [0-1] vs 0 [0-1]; *P* = .01) after delivery; reduced depressive symptoms (RR, 0.73; 95% CI, 0.54-0.98; *P* = .01); and increased breastfeeding milk volume measured at 28.5, 52.5, and 76.5 hours after delivery. No adverse events occurred in either of the 2 groups.

**Conclusions and Relevance:**

Results of this trial showed that acupoint hot compress after vaginal delivery decreased postpartum urinary retention, uterine contraction pain, and depressive symptoms and increased breastfeeding milk volume. Acupoint hot compress may be considered as an adjunctive intervention in postnatal care that meets patient self-care needs.

**Trial Registration:**

Chinese Clinical Trial Registry Identifier: ChiCTR2000038417

## Key Points

**Question:**

Does acupoint hot compress in the abdominal, lumbosacral, and plantar regions lower the incidence of urinary retention, decrease uterine contraction pain, lessen depressive symptoms, and improve lactation after vaginal delivery?

**Findings:**

In this randomized clinical trial involving 1200 postpartum individuals in China, acupoint hot compress resulted in a significant decrease in postpartum urinary retention incidence, uterine contraction pain, and depressive symptoms and an increase in breastfeeding milk volume.

**Meaning:**

Findings of this trial suggest that acupoint hot compress could be considered as an adjunctive intervention in postnatal care.

## Teaser

This randomized clinical trial evaluates urinary retention, uterine contraction pain, depressive symptoms, and lactation after acupoint hot compress treatment in women during their early postpartum period.

## Introduction

The postpartum period, especially the first few weeks, is a challenging time for mothers to adjust to the situation of having newborns. The World Health Organization guidelines for postnatal care recommend that mothers and newborns receive care within 24 hours after birth.^1^ Moreover, patient self-care needs, even in the first few days after delivery, go beyond physical health and also extend to emotional well-being.^2^ The underlying mechanism of acupoint hot compress for individuals during the early postpartum period may be the promotion of local blood circulation, improvement of tissue metabolism and peripheral hemodynamics, inhibition of sympathetic nerve activity, and induction of parasympathetic nerve activity dominance.^3,4,5^

The hot compress has proven to be an effective complementary treatment for myofascial pain syndrome in the upper trapezius muscle^6^ and has exhibited an extraordinary capability for regulating the pressure pain threshold and improving the quality of life.^7^ Warm compress bistage intervention has been found to substantially reduce the intensity of pain on the day after delivery.^8^ Acupoint hot compress, a combination of acupoints and natural physical agent heat, is more acceptable than other treatments both physically and mentally for patients and their families during puerperium because of its noninvasive feature. However, the effects of acupoint hot compress during the early postpartum period remain uncertain because of the lack of evidence-based support from clinical trials.

The main objective of this randomized clinical trial was to assess whether acupoint hot compress involving the abdominal, lumbosacral, and plantar regions could reduce the incidence of postpartum urinary retention, relieve postpartum uterine contraction pain, prevent emotional disorders, and promote lactation.

## Methods

This multicenter randomized clinical trial was conducted at 12 hospitals in China (Women’s Hospital, School of Medicine, Zhejiang University, Hangzhou; Tongde Hospital of Zhejiang Province, Hangzhou; Yiwu Maternity and Child Health Care Hospital, Yiwu; Jiaxing Maternity and Child Health Care Hospital, Jiaxing; Zhejiang Xiaoshan Hospital, Hangzhou; Zhoushan Women and Children Hospital, Zhoushan; Shaoxing Maternity and Child Health Care Hospital, Shaoxing; The Women and Children Hospital of Dongyang, Dongyang; Ruian People's Hospital, Ruian; Cixi Maternity and Child Health Care Hospital, Cixi; Wenling Maternity and Child Health Care Hospital, Wenling; and Xianju People's Hospital, Xianju). The institutional review board at each site approved the trial protocol (Supplement 1).^9^ Written informed consent was obtained from each participant. We followed the Consolidated Standards of Reporting Trials (CONSORT) reporting guideline.

### Participants

Pregnant patients were recruited at each hospital using an announcement poster. Between January 17 and August 15, 2021, these individuals were screened for eligibility and enrolled. Inclusion criteria were older than 18 years, primiparity with a singleton pregnancy, gestational age of 37 to 42 weeks, and breastfeeding after delivery. The main exclusion criteria were family history of mental illness or tumor; mental or psychological disorders, traumatic events, or communication barriers in the past 3 years; prepregnancy central nervous system diseases, internal and surgical diseases, breast dysplasia, urogenital-related diseases, infectious diseases in the past 18 months, and long-term drug treatment; skin damage, ulceration, sensory disorders, acute closed injury, suppurative infection, acute inflammation and other infectious diseases, skin diseases, severe diabetes, and high fever associated with an allergy to the product materials; and incomplete information that was required by the trial.

Participants’ self-reported race and ethnicity data were routinely collected without ethnic discrimination. The race and ethnicity identified by individuals included Han and minority groups. Data collection was completed on August 18, 2021.

### Randomization

**Figure 1.**
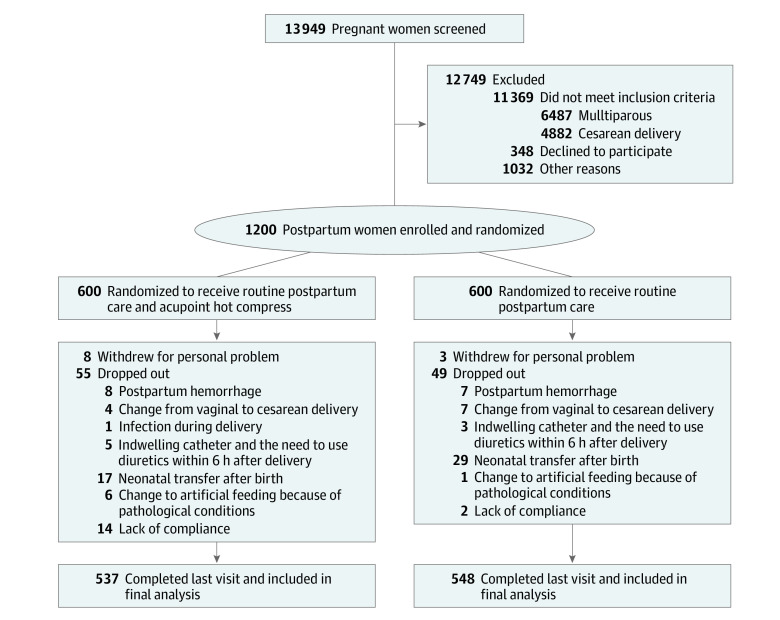
Profile of the Randomized Clinical Trial

After vaginal delivery, eligible participants were randomized 1:1 to the intervention group (receiving routine postpartum care and acupoint hot compress) or the control group (receiving routine postpartum care) (Figure 1). The randomization number sequence was performed by an independent statistician (J. Zhou) using R, version 4.1.0 (R Foundation for Statistical Computing), with a block size of 4. Randomization was stratified by each site and performed by an independent researcher (including Y.W., W.J., L.Z., W.W., C.Z., S. Lu., R.W., L.J., H.G., J. Zhang, J.L., and G.S.) at each site who was not involved in the outcome assessment. The outcomes assessors were blinded to the treatment randomization. Although the trial was open label, the statisticians who were blinded to the randomization were responsible for analyzing the data.

### Interventions

Participants in the control group received routine postpartum care, including observation of their vital signs (blood pressure, heart rate, respiratory rate, temperature, and oxygen saturation of blood), monitoring of the amount of vaginal bleeding and discharge of lochia, palpation of their fundus, cleansing of their vulva, and an early interaction with their newborns.^10^

**Figure 2.**
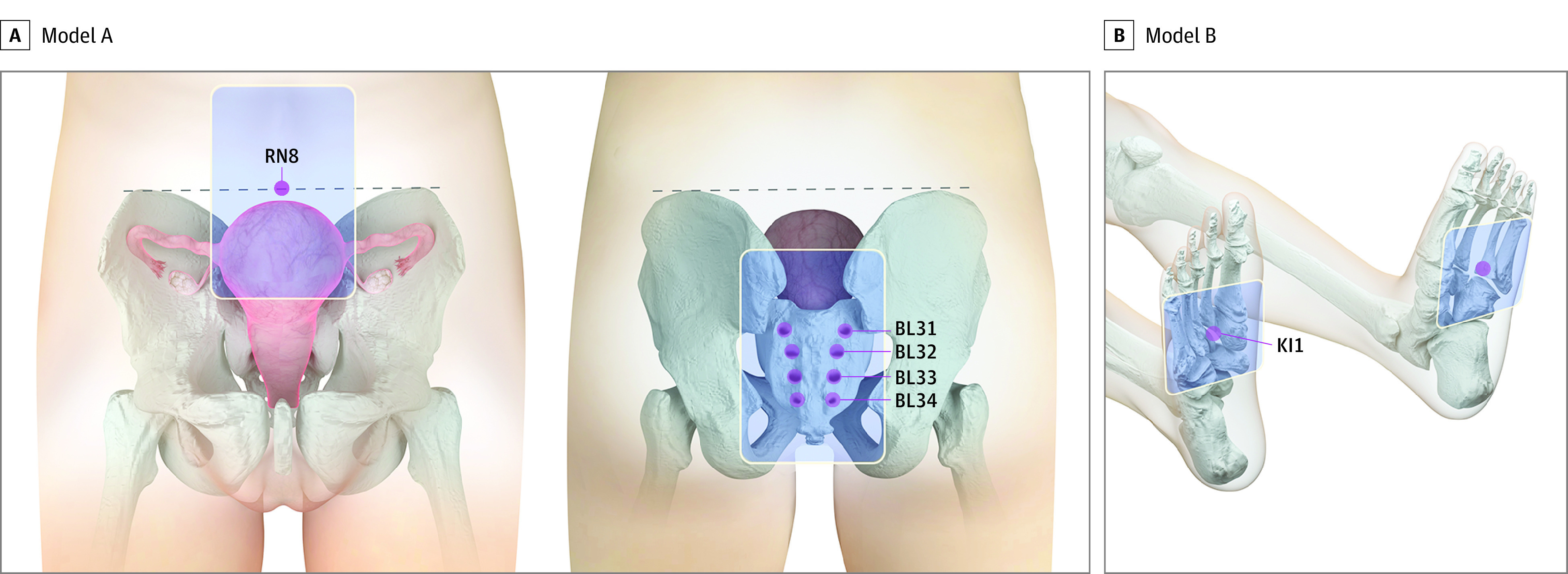
Location of the Acupoints Used for the Intervention Group Shenque (RN8 acupoint) is in the center of the umbilicus. Baliao (BL31-BL34 acupoints) is in the region of the sacrum between the posterior-superior iliac spine and the posterior midline in the first (Shangliao), second (Ciliao), third (Zhongliao), and fourth (Xialiao) posterior sacral foramen. Yongquan (KI1 acupoint) is on the sole in the depression when the foot is in plantar flexion and in the anterior depression when the foot is flexed, approximately at the junction of the anterior one-third and posterior two-thirds of the sole.

Participants in the intervention group received routine postpartum care along with a 4-hour acupoint hot compress at a constant mean (SD) temperature of 45 (2) °C within 30 minutes after delivery (time point 1), 24 hours after delivery (time point 2), and 48 hours after delivery (time point 3). As shown in Figure 2, at time point 1, 2 hot cores (model A) were administered on Shenque (RN8 acupoint) in the center of the umbilicus and on Baliao (BL31-BL34 acupoints) in the region of the sacrum between the posterior-superior iliac spine and the posterior midline in the first (Shangliao), second (Ciliao), third (Zhongliao), and fourth (Xialiao) posterior sacral foramen. Under model B, 2 hot cores were bilaterally administered on Yongquan (KI1 acupoint) on the sole in the depression when the foot was in plantar flexion and in the anterior depression when the foot was flexed, approximately at the junction of the anterior one-third and posterior two-thirds of the sole. For time points 2 and 3, only 1 hot core (model A) was administered on Shenque.

The locations of the involved acupoints were described according to the Nomenclature and Location of Acupuncture Points of the 2006 National Standard of People’s Republic of China. The hot compress was applied using a licensed class II medical device (Hu-Chao-Nuan-Gong-Bao; Jiangxi Shenghe Industrial Development Co). Four hot cores in model A (sized 11.5 × 8 cm, with an entire patch of 13 × 10 cm) and another 2 hot cores in model B (sized 8 × 7 cm, with an entire patch of 16 × 9 cm) were included in the licensed device. The duration of inpatient hospital stay was 3 days after the vaginal delivery.

### Outcomes Measures

The primary outcome was the incidence of postpartum urinary retention. Postpartum urinary retention was defined as the first urination occurring more than 6.5 hours after delivery and/or use of an indwelling catheter within 72 hours after delivery. The time of the first urination after delivery and whether an indwelling catheter was used within 72 hours after delivery were recorded.

The first secondary outcome was postpartum uterine contraction pain intensity. For this outcome, the visual analog scale (VAS) was used with a horizontal ruler of 100 mm; the VAS score ranged from 0 (indicating no pain) to 100 (indicating unbearable pain). Pain intensity was measured at 6.5, 28.5, 52.5, and 76.5 hours after delivery.

The second secondary outcome was depressive symptoms. For this outcome, the Edinburgh Postnatal Depression Scale (EPDS) was used to screen for depressive symptoms at 76.5 hours after delivery. The EPDS contains 10 items: pessimism, lack of interest, self-blame, worry, fear, impaired ability, sleep disorder, sadness, tearfulness, and self-injury or suicidal ideation. Pessimism, lack of interest, and worry were scored from 0 (indicating no symptoms) to 3 (indicating most severe symptoms). Self-blame, fear, impaired ability, sleep disorder, sadness, tearfulness, and self-injury or suicidal ideation were reverse-scored.

The third secondary outcome was lactation. For this outcome, the lactation initiation time was recorded, and the breastfeeding milk volume was measured at 28.5 hours, feeding mood and times were measured at 52.5 hours, and newborn weight was measured at 76.5 hours after delivery. The breastfeeding milk volume was scored by an on-site observer and measured as follows: no milk outflow during finger extrusion (–), milk flows out occasionally during finger extrusion and cannot meet the needs of the newborn (+), milk flows out slowly during finger extrusion but meets the needs of the newborn (+ +), milk flows out continuously during finger extrusion and meets the needs of the newborn (+ + +), and milk flows out in a jet stream during finger extrusion and meets the needs of the newborn (+ + + +).

### Adverse Events

Adverse events were documented throughout the trial. On the basis of the potential association of an adverse event with the acupoint hot compress procedure, on-site specialists categorized the adverse events as either treatment related (including local skin allergy, pruritus, pain, scald, ulceration, subcutaneous hemorrhage, hematoma, and local infection) or nontreatment related within 2 hours of occurrence.

### Statistical Analysis

According to the results of the pilot study, the incidence rate of postpartum urinary retention was 7.7% in the intervention group and 25.7% in the control group. Assuming a 1:1 ratio of the intervention group to the control group and using a 1-sided test, a significance level of α = .025 and a power (1-β) of 80% were established. We estimated that 1200 participants were required for the trial, assuming that 20% of participants might drop out of the study.

Baseline characteristics and clinical outcomes were described on the basis of the per-protocol population (n = 1085). Continuous variables were presented as a mean (SD) or median (IQR) according to the distribution of variables, and categorical variables were described as numbers and percentages.

For the primary outcome, the incidences of postpartum urinary retention in the intervention and control groups were compared using the Fisher exact test, and the relative risk (RR) and corresponding 95% CIs were calculated. For the secondary outcomes, the postpartum uterine contraction pain intensity levels at 6.5, 28.5, 52.5, and 76.5 hours after delivery in the 2 groups were compared using the Wilcoxon rank sum test. Depressive symptoms were compared using the Fisher exact test, and the RR and corresponding 95% CIs were calculated. Lactation initiation time was compared using the Wilcoxon rank sum test. The Fisher exact test was used to compare the breastfeeding milk volume as well as feeding mood and times assessed at 28.5, 52.5, and 76.5 hours after birth between the 2 groups. Newborn weight at 28.5, 52.5, and 76.5 hours after delivery were analyzed using an unpaired, 2-tailed *t* test. To consider the potential effects of spinal analgesia on the outcomes, we conducted a subgroup analysis.

Statistical analysis was based on per-protocol population and was performed by 2 independent analysts (Y.L. and M.T.) using R, version 4.1.0 (R Foundation for Statistical Computing). A 2-sided *P* < .05 was considered to be statistically significant.

## Results

A total of 1200 participants were randomized to the intervention group (n = 600) or the control group (n = 600). Among the randomized participants, 1085 completed the study, of whom 537 (89.5%) were in the intervention group and 548 (91.3%) were in the control group (Figure 1). These participants had a median (IQR) age of 26.0 (24.0-29.0) years. Most baseline characteristics were similar between the 2 groups except for educational level (Table 1). The intervention group had a median (IQR) predelivery body mass index (BMI) of 26.04 (23.88-28.04), and the control group had a median (IQR) predelivery BMI of 26.10 (24.24-27.92). No adverse events occurred in either of the 2 groups during the study.

**Table 1.** Baseline Characteristics of the Participants

Characteristic	No. (%)	
	Intervention group (n = 537)	Control group (n = 548)
Age, median (IQR), y	26.00 (24.00-29.00)	26.00 (24.00-29.00)
Prepregnancy BMI, median (IQR)	20.07 (18.67-22.27)	20.41 (18.89-22.06)
Place of residence		
Urban	262 (48.8)	241 (44.0)
Suburban	172 (32.0)	200 (36.5)
Rural	103 (19.2)	107 (19.5)
Educational level^a^		
Graduate	23 (4.3)	20 (3.6)
Undergraduate	285 (53.1)	252 (46.0)
College	42 (7.8)	48 (8.8)
Secondary vocational	78 (14.5)	121 (22.1)
General higher secondary	109 (20.3)	107 (19.5)
Occupation category^b^		
Principal state ministries, party group organizations, enterprises, and institutions	2 (0.4)	1 (0.2)
Professional technologists	72 (13.4)	54 (9.9)
Affair management and related jobs	12 (2.2)	10 (1.8)
Business services	232 (43.2)	241 (44.0)
Agriculture, forestry, stock breeding, fisheries, and irrigation	56 (10.4)	43 (7.8)
Production, transportation, equipment operation, and related services	6 (1.1)	3 (0.5)
Unemployed	157 (29.2)	196 (35.8)
Mode of conception		
Natural	519 (96.6)	537 (98.0)
Ovarian stimulation	5 (0.9)	2 (0.4)
Artificial insemination	2 (0.4)	2 (0.4)
IVF, ICSI, or PGT	11 (2.0)	7 (1.3)
Pregnancy complications^c^		
Yes	135 (25.1)	129 (23.5)
No	402 (74.9)	419 (76.5)
Fetus and appendages		
Abnormal^d^	15 (2.8)	11 (2.0)
Normal	522 (97.2)	537 (98.0)
Gestational age of delivery, median (IQR), wk	39.00 (38.00-40.00)	39.00 (39.00-40.00)
Predelivery BMI, median (IQR)	26.04 (23.88-28.04)	26.10 (24.24-27.92)
Labor induction^e^		
Yes	188 (35.0)	191 (34.9)
No	349 (65.0)	357 (65.1)
Augmentation by oxytocin		
Yes	282 (52.5)	267 (48.7)
No	255 (47.5)	281 (51.3)
Rupture of membranes		
Spontaneous	286 (53.3)	321 (58.6)
Artificial	251 (46.7)	227 (41.4)
Spinal analgesia for labor pain		
Yes	437 (81.4)	423 (77.2)
No	100 (18.6)	125 (22.8)
Duration of labor stages, median (IQR)		
First	405.00 (286.00-560.00)	392.50 (269.75-570.00)
Second	52.00 (34.00-87.00)	49.00 (32.75-78.25)
Third	7.00 (5.00-8.00)	7.00 (5.00-8.00)
Total	485.00 (350.00-648.00)	475.00 (325.00-635.00)
Assisted vaginal delivery^f^		
Yes	13 (2.4)	13 (2.4)
No	524 (97.6)	535 (97.6)
Newborn weight, median (IQR), g	3280.00 (3050.00-3510.00)	3250.00 (3030.00-3500.00)
Newborn length, median (IQR), cm	50.00 (50.00-50.00)	50.00 (50.00-50.00)
Apgar score, median (IQR)		
1 min	10.00 (10.00-10.00)	10.00 (10.00-10.00)
5 min	10.00 (10.00-10.00)	10.00 (10.00-10.00)
Time between birth and early sucking of newborns, median (IQR), min	30.00 (21.00-37.00)	30.00 (20.75-38.00)

Abbreviations: BMI, body mass index (calculated as weight in kilograms divided by height in meters squared); IVF, in vitro fertilization; ICSI, intracytoplasmic sperm injection; PGT, preimplantation genetic testing.

^a^ Categories according to the 2007 Codes for Record of Formal Schooling of the People’s Republic of China.

^b^ Categories according to the 2015 National Occupational Classification Code of the People’s Republic of China.

^c^ Included hypertensive disorders of pregnancy, intrahepatic cholestasis of pregnancy, gestational diabetes, hypothyroidism, Hashimoto thyroiditis, antiphospholipid syndrome, hyperlipidemia in pregnancy, hydronephrosis in pregnancy, obesity, and anemia.

^d^ Included macrosomia, fetal growth restriction, and oligohydramnios.

^e^ Included a vaginal dinoprostone slow-release system, balloon catheter, and oxytocin.

^f^ Included forceps-assisted and vacuum-assisted vaginal delivery.

### Primary Outcome

The effects of acupoint hot compress were evident in these results. As shown in Table 2, the incidence of postpartum urinary retention in 24 of 537 participants (4.5%) in the intervention group was significantly lower than that in 42 of 548 participants (7.7%) in the control group (RR, 0.58; 95% CI, 0.35-0.98; *P* = .03). As outlined in Table 3, among participants who received spinal analgesia, those in the intervention group had a significantly decreased incidence of postpartum urinary retention compared with those in the control group (20 of 437 [4.6%] vs 36 of 423 [8.5%]; RR, 0.54; 95% CI, 0.31-0.95; *P* = .03). No significant difference was found between participants in the intervention and control groups who did not undergo spinal analgesia (4 of 100 [4.0%] vs 6 of 125 [4.8%]; RR, 0.83; 95% CI, 0.23-3.04; *P* > .99), suggesting no effects from the acupoint hot compress application.

**Table 2.** Primary and Secondary Outcomes

Variable	No. (%)		RR (95% CI)	*P* value^**a**^
	Intervention group (n = 537)	Control group (n = 548)		
Primary outcome				
Postpartum urinary retention				
Yes	24 (4.5)	42 (7.7)	0.58 (0.35-0.98)	.03
No	513 (95.5)	506 (92.3)		
Secondary outcomes				
Postpartum uterine contraction pain assessed with VAS at different time points, median (IQR) score^b^				
6.5 h	1 (1-2)	2 (1-2)	NA	<.001
28.5 h	1 (0-1)	1 (1-2)	NA	<.001
52.5 h	1 (0-1)	1 (0-1)	NA	<.001
76.5 h	0 (0-1)	0 (0-1)	NA	.01
Depression levels screened with EPDS, score^c^				
<9	446 (83.1)	420 (76.6)	0.73 (0.54-0.98)	.01
≥9	91 (16.9)	128 (23.4)		

Abbreviations: EPDS, Edinburgh Postnatal Depression Scale; NA, not applicable; RR, relative risk; VAS, visual analog scale.

^a^ All tests were 2-sided. *P* < .05 was considered to be significant.

^b^ Calculated using Wilcoxon rank sum test. The VAS score ranged from 0 (indicating no pain) to 100 (indicating unbearable pain).

^c^ Calculated using Fisher exact test. Cutoff value was based on the 2021 Experts Consensus on Screening and Diagnosis of Perinatal Depression of the People’s Republic of China. The EPDS contains 10 items: pessimism, lack of interest, self-blame, worry, fear, impaired ability, sleep disorder, sadness, tearfulness, and self-injury or suicidal ideation. Pessimism, lack of interest, and worry were scored from 0 (indicating no symptoms) to 3 (indicating most severe symptoms). Self-blame, fear, impaired ability, sleep disorder, sadness, tearfulness, and self-injury or suicidal ideation were reverse scored.

**Table 3.** Subgroup Analysis for Participants With or Without Spinal Analgesia for Labor Pain

Variable	No. (%)		RR (95% CI)	*P* value^**a**^
	Intervention group (n = 437 with spinal analgesia)	Control group (n = 423 with spinal analgesia)		
Primary outcome				
Postpartum urinary retention^b^				
Yes	20 (4.6)	36 (8.5)	0.54 (0.31-0.95)	.03
No	417 (95.4)	387 (91.5)		
Secondary outcomes				
Postpartum uterine contraction pain assessed with VAS at different time points, median (IQR) score^c^				
6.5 h	1 (1-2)	2 (1-2)	NA	<.001
28.5 h	1 (0-1)	1 (1-2)	NA	.004
52.5 h	1 (0-1)	1 (0-1)	NA	.006
76.5 h	0 (0-1)	0 (0-1)	NA	.03
Depression levels screened with EPDS, score^b^^,^^d^				
<9	361 (82.6)	327 (77.3)	0.77 (0.55-1.07)	.06
≥9	76 (17.4)	96 (22.7)		

Abbreviations: EPDS, Edinburgh Postnatal Depression Scale; NA, not applicable; RR, relative risk; VAS, visual analog scale.

^a^ All tests were 2-sided. *P* < .05 was considered significant.

^b^ Calculated using Fisher exact test.

^c^ Calculated using Wilcoxon rank sum test. The VAS score ranged from 0 (indicating no pain) to 100 (indicating unbearable pain).

^d^ Cutoff value was based on the 2021 Experts Consensus on Screening and Diagnosis of Perinatal Depression of the People’s Republic of China. The EPDS contains 10 items: pessimism, lack of interest, self-blame, worry, fear, impaired ability, sleep disorder, sadness, tearfulness, and self-injury or suicidal ideation. Pessimism, lack of interest, and worry were scored from 0 (indicating no symptoms) to 3 (indicating most severe symptoms). Self-blame, fear, impaired ability, sleep disorder, sadness, tearfulness, and self-injury or suicidal ideation were reverse scored.

### Secondary Outcomes

As shown in Table 2, the postpartum uterine contraction pain intensity of the intervention group compared with the control group was significantly alleviated when measured at 6.5 hours (median [IQR] VAS score, 1 [1-2] vs 2 [1-2]; *P* < .001), 28.5 hours (median [IQR] VAS score, 1 [0-1] vs 1 [1-2]; *P* < .001), 52.5 hours (median [IQR] VAS score, 1 [0-1] vs 1 [0-1]; *P* < .001), and 76.5 hours (median [IQR] VAS score, 0 [0-1] vs 0 [0-1]; *P* = .01) after delivery. Among participants who received spinal analgesia, acupoint hot compress significantly alleviated postpartum uterine contraction pain at all 4 time points in those in the intervention group vs the control group (median [IQR] VAS score: 6.5 hours, 1 [1-2] vs 2 [1-2], *P* < .001; 28.5 hours, 1 [0-1] vs 1 [1-2], *P* = .004; 52.5 hours, 1 [0-1] vs 1 [0-1], *P* = .006; 76.5 hours, 0 [0-1] vs 0 [0-1], *P* = .03) (Table 3). Participants in the intervention group vs those in the control group who did not undergo spinal analgesia also had improvement in pain when it was measured at 28.5 hours (median [IQR] VAS score, 1 [0-1] vs 1 [1-2]; *P* = .02) and 52.5 hours (median [IQR] VAS score, 0 [0-1] vs 1 [0-1]; *P* = .02) after delivery.

As shown in Table 2, acupoint hot compress significantly decreased the incidence of depression symptoms in participants in the intervention group vs the control group (<9 EPDS score, 446 [83.1%] vs 420 [76.6%]; RR, 0.73; 95% CI, 0.54-0.98; *P* = .01). As outlined in Table 3, participants who received spinal analgesia in the intervention group had a significant decrease in the incidence of depressive symptoms compared with participants in the control group (<9 EPDS score, 361 [82.6%] vs 327 [77.3%]; RR, 0.77; 95% CI, 0.55-1.07; *P* = .06). Participants in the intervention group vs the control group without spinal analgesia also had reduced incidence of depressive symptoms (<9 EPDS score, 85 [85.0%] vs 93 [74.4%]; RR, 0.59; 95% CI, 0.30-1.16; *P* = .07).

The breastfeeding milk volume of the intervention group compared with the control group was significantly increased when measured at 28.5, 52.5, and 76.5 hours after delivery. Among participants who received spinal analgesia, those in the intervention group vs those in the control group had a significantly increased breastfeeding milk volume when measured at 28.5 hours and 76.5 hours after delivery. Participants without spinal analgesia in the intervention group vs the control group also had higher milk volume when measured at 52.5 hours and 76.5 hours after delivery.

As outlined in eTable 1 and eTable 2 in Supplement 2, there were no significant differences between the 2 groups in lactation initiation time, feeding mood, feeding times, or newborn weight. The effects of acupoint hot compress were nonexistent when receipt of spinal analgesia was considered.

## Discussion

The present trial found that, after a vaginal delivery, participants benefited from acupoint hot compress involving the abdominal, lumbosacral, and plantar regions. These individuals had a lower incidence of urinary retention, attenuated uterine contraction pain, alleviated depressive symptoms, and increased breastfeeding milk volume.

As a common complication in women during the immediate postpartum period, the prevalence of postpartum urinary retention ranges from 1.5% to 45% according to varying diagnostic criteria.^11^ However, the routine measurement of postpartum urinary retention has not been established in obstetrics,^11,12^ and the lack of guidelines is one of the major problems in treating individuals with postpartum urinary retention.^11,13,14^ As a result, we chose postpartum urinary retention as the primary outcome in the present trial, which may provide evidence for clinical practice and future guidelines. Any misdiagnosis or delay in the diagnosis of postpartum urinary retention can cause bladder overdistension, leading to irreversible detrusor damage.^15^ We found that acupoint hot compress significantly decreased the incidence of postpartum urinary retention for trial participants.

The independent risk factors for postpartum urinary retention include instrumental delivery and the absence of spontaneous voiding before leaving the delivery room, no intact perineum and vulvar edema or perineal hematoma, race or ethnicity other than White, nulliparity, a BMI lower than 30 at the end of pregnancy, meconium-stained amniotic fluid, a nonoperative vaginal birth, vacuum extraction, a pushing stage longer than 60 minutes, and a perineal tear.^16,17^ In this trial, the participants presented with some of these risk factors before delivery. For example, the median predelivery BMI was 26.04 for the intervention group and 26.10 for the control group. Consequently, acupoint hot compress is promising in pregnant individuals with risk factors.

As perinatal depressive symptoms are associated with oppositional defiant disorder in offspring, early identification and management of prenatal and postnatal depression are important.^18^ In this trial, we screened depressive symptoms at 76.5 hours after delivery because mood disturbances at day 3 in the postpartum period are the best predictors of depression occurring at week 6 of the postpartum period.^19^ Postpartum depression is a widely recognized public health concern that has important implications for the well-being of new mothers, their infants, and their families.^20,21^ In 1 study, the pooled prevalence of postpartum depression was 14.8%, and there has been an increasing pattern in the past decade.^22^ Although numerous mechanisms that mediate the development of postpartum depression have been found,^23^ the cause of postpartum depression has not been fully elucidated. Some women who breastfeed may even be unwilling to take pharmacological treatments out of concern that antidepressant medication can seep into breast milk and potentially expose their infants.^24^ Dietary quality is a modifiable risk factor for depression because some nutritional factors may modulate the potential biological pathways, including inflammation, oxidative stress, gut microbiome, epigenetic modifications, and neuroplasticity.^25^ Poor dietary quality or an imbalanced diet has been associated with postpartum depression in lactating Chinese women.^26,27^ In the present trial, no significant differences were found in the postpartum diet or appetite between the intervention and control groups, suggesting that it was acupoint hot compress that significantly decreased the depression levels in these participants.

Low occurrence of breastfeeding and low birth weight were shown to be risk factors for postpartum depression.^28^ Although breastfeeding can protect individuals from postpartum depression, it was not found to be associated with incomplete breastfeeding in some studies, and there were other risk factors for postpartum depression.^29^ In the early postpartum period, mothers and infants navigate a critical neuroendocrine transition from pregnancy to lactation, and the shared neuroendocrine mechanism is hypothesized to contribute to both lactation failure and perinatal mood disorders.^30^ In this trial, we found that acupoint hot compress significantly increased the breastfeeding milk volume when measured at 28.5, 52.5, and 76.5 hours after delivery, and depressive symptoms were significantly decreased when measured at 76.5 hours.

### Limitations

This study has several limitations. First, because there was no available placebo hot compress, the participants were not blinded to the group randomization. Second, because the hot compress may cover other acupoints in the location, active control participants using these acupoints should be included in further study. Third, we used a standard treatment regimen to evaluate the efficacy of acupoint hot compress on early puerperal rehabilitation among participants after a vaginal delivery. We did not use personalized intervention protocols based on different syndromes of the participants (Bian Zheng method), which might have caused bias. Fourth, all participants were healthy nulliparas with a singleton pregnancy at baseline; thus, this finding might not be generalized to individuals with complications or multiparas. Fifth, the volume of postpartum residual urine was not measured with ultrasonographic examination. However, we used a broader definition to identify postpartum urinary retention, which may partly address this issue. Sixth, as there are no widely recognized criteria to assess breastfeeding milk volume, the measurement we used may lead to some bias. Seventh, as all interventions and measurements were conducted during hospitalization after a vaginal delivery, the long-term effects of the acupoint hot compress are unknown. Further work is needed to investigate the long-term efficacy and the mechanism underlying the action of this intervention.

## Conclusions

This randomized clinical trial found that acupoint hot compress applied to the abdominal, lumbosacral, and plantar regions decreased the incidence of postpartum urinary retention, reduced uterine contraction pain, improved depressive symptoms, and increased breastfeeding milk volume for individuals after a vaginal delivery. Based on these results, acupoint hot compress may be considered as an adjunctive intervention in postnatal care that meets patients’ self-care needs.

## Notes

Supplement 1.
Trial Protocol

Supplement 2. **eTable 1.** Secondary Analysis Lactation **eTable 2.** Subgroup Analysis for Participants With or Without Spinal Analgesia Lactation

Supplement 3.
Data Sharing Statement
