# Supplementary material for: Effect of Acupoint Hot Compress on Postpartum Urinary Retention After Vaginal Delivery: A Randomized Clinical Trial
Source: JAMA Netw Open. 2022 May 23;5(5):e2213261. doi: 10.1001/jamanetworkopen.2022.13261 (PMC9127553; doi:10.1001/jamanetworkopen.2022.13261)
Supplement: Supplement 1. — Trial Protocol [file jamanetwopen-e2213261-s001.pdf]

Supplement 1 to

Effects of acupoint hot compress during early puerperium on parturients after a vaginal delivery: a randomized clinical trial

This supplement contains the following items:

1. Final protocol, summary of changes from the published original protocol.
2. Final statistical analysis plan, summary of changes from the published original protocol.

Effects of acupoint hot compress during early puerperium on parturients after a vaginal delivery: study protocol for a randomized clinical trial

Clinical sites:

Women's Hospital, School of Medicine, Zhejiang University
Tongde Hospital of Zhejiang Province
Yiwu Maternity and Child Health Care Hospital
Jiaxing Maternity and Child Health Care Hospital
Zhejiang Xiaoshan Hospital
Zhoushan Women and Children Hospital
Shaoxing Maternity and Child Health Care Hospital
The Women & Children Hospital of Dongyang
Ruian People's Hospital
Cixi Maternity and Child Health Care Hospital
Wenling Maternity and Child Health Care Hospital
Xianju People's Hospital

Data Management and Statistical Centers:

Women's Hospital, School of Medicine, Zhejiang University

Data:

Original protocol date: July 16, 2020
Amendment date: August 25, 2020

Confidentiality Statement

This document is the intellectual property of the Investigators. The information provided in this document is strictly confidential and is available for review to the sponsor, investigators, potential investigators, appropriate Ethics Committees, Investigational Review Boards, and other government regulatory bodies. No disclosure should take place without written authorization from the protocol developing investigators, except to the extent necessary needed to obtain informed consent from potential subjects.

Table of Contents

1. Study Contact and Organization 4
2. Study Design 9
  2.1 Study Overview 9
  2.2 Background 9
  2.3 Materials and methods 10
    2.3.1 Objectives 10
    2.3.2 Trial registration 10
    2.3.3 Trial design 10
    2.3.4 Recruitment 12
    2.3.5 Participants 12
      2.3.5.1 Inclusion criteria 12
      2.3.5.2 Exclusion criteria 12
      2.3.5.3 Subject withdrawal 13
      2.3.5.4 Subject drop-out and discontinuation 13
  2.4 Sample size calculation 13
  2.5 Randomization and blinding 13
  2.6 Interventions 13
    2.6.1 Control group 13
    2.6.2 Intervention group 13
  2.7 Baseline data 15
  2.8 Outcomes measures 16
    2.8.1 Primary outcome 16
    2.8.2 Secondary outcomes 16
  2.9 Safety Evaluation 16
3. Informed Consent 16
  Notification Page 16
  Signature Page 19
4. Risks 20
5. Benefits 20
6. Data Management 21
7. Statistical analysis 21
8. Privacy 21
9. Funding 21
10. Reference 23
11. Update on the Published Protocol 23

1. Study Contact and Organization

1.1 Study Contacts

Principal Investigator for grant

Fan Qu, MD, PhD
Women's Hospital, School of Medicine, Zhejiang University
No.1 Xueshi Road, Hangzhou, Zhejiang, 310006, China
Phone: +86 13429119143
E-mail: syqufan@zju.edu.cn

Principal Investigator for trial

Yuhang Zhu, MS
Women's Hospital, School of Medicine, Zhejiang University
No.1 Xueshi Road, Hangzhou, Zhejiang, 310006, China
Phone: +86 15267047921
E-mail: 11918383@zju.edu.cn

1.2 Recruiting Sites

Women's Hospital, School of Medicine, Zhejiang University

Yuhang Zhu, MS
No.1 Xueshi Road, Hangzhou, Zhejiang, 310006, China
Phone: +86 15267047921
E-mail: 11918383@zju.edu.cn

Fangfang Wang, PhD
No.1 Xueshi Road, Hangzhou, Zhejiang, 310006, China
Phone: +86 13805783291
E-mail: drwangfangfang@zju.edu.cn

Xiaoli Hu, MS, RN
No.1 Xueshi Road, Hangzhou, Zhejiang, 310006, China
Phone: +86 571 87061501
E-mail: huxiaoli@zju.edu.cn

Dongmei Ma, RN
No.1 Xueshi Road, Hangzhou, Zhejiang, 310006, China
Phone: +86 571 87061501
E-mail: madm@zju.edu.cn

Chunxiao Hu, RN

No.1 Xueshi Road, Hangzhou, Zhejiang, 310006, China
Phone: +86 571 87061501
E-mail: huchunxiao12345@163.com

Yan Wu, MS
No.1 Xueshi Road, Hangzhou, Zhejiang, 310006, China
Phone: +86 571 87061501
E-mail: withoutsalt@zju.edu.cn

Xuan Yang, BS
No.1 Xueshi Road, Hangzhou, Zhejiang, 310006, China
Phone: +86 571 87061501
E-mail: yangxuan@zju.edu.cn

Fang Wang, MS, RN
No.1 Xueshi Road, Hangzhou, Zhejiang, 310006, China
Phone: +86 571 87061501
E-mail: fangw@zju.edu.cn

Xinfen Xu, BS, RN
No.1 Xueshi Road, Hangzhou, Zhejiang, 310006, China
Phone: +86 571 87061501
E-mail: xuxinf@zju.edu.cn

Tongde Hospital of Zhejiang Province
Wei Wang, RN
No. 234 Gucui Road, Hangzhou,Zhejiang,310012, China
Phone: +86 15824166333
E-mail: ww1455610241@163.com

Meiyuan Jin, BS
No. 234 Gucui Road, Hangzhou,Zhejiang,310012, China
Phone: +86 13732229339
E-mail: jmy8803@126.com

Yuefang Zhou, RN
No. 234 Gucui Road, Hangzhou,Zhejiang,310012, China
Phone: +86 13958108547
E-mail: zyf1973@163.com

Yiwu Maternity and Child Health Care Hospital

Lianqing Gong, RN
No.C100 Xinke Road, Yiwu, Zhejiang,322000, China
Phone: +86 13616798556
E-mail: ywfbhlb@163.com

Jinhe Huang, RN
No.C100 Xinke Road, Yiwu, Zhejiang,322000, China
Phone: +86 13757907773
E-mail: hjh1005679232@163.com

Liuyan Zhong, RN
No.C100 Xinke Road, Yiwu, Zhejiang,322000, China
Phone: +86 15205795070
E-mail: liuyan19911213@163.com

Jiaxing Maternity and Child Health Care Hospital

Shuiqin Gu, MS, RN
No.2468 East Zhonghuan Road, Jiaxing, Zhejiang, 314000, China
Phone: +86 13967367217
E-mail: jxfbyhlb@126.com

Jing Jin, RN
No.2468 East Zhonghuan Road, Jiaxing, Zhejiang, 314000, China
Phone: +86 13736826526
E-mail: jj13736806526@163.com

Wei Jiang, RN
No.2468 East Zhonghuan Road, Jiaxing, Zhejiang, 314000, China
Phone: +86 13736429454
E-mail: jw13736429454@163.com

Zhejiang Xiaoshan Hospital

Honglou Chen, RN
NO. 728 North Yucai Road, Hangzhou, Zhenjiang, 311202, China
Phone: +86 18967168605
E-mail: chen.honglou@163.com

Guofang Shen, RN
NO. 728 North Yucai Road, Hangzhou, Zhenjiang, 311202, China
Phone: +86 18967168798
E-mail: xyfbksgf@163.com

Jinfang Kong, RN
NO. 728 North Yucai Road, Hangzhou, Zhenjiang, 311202, China
Phone: +86 18967168596
E-mail: 18967168596@163.com

<u>Zhoushan Women and Children Hospital</u>

Yan He, RN
NO.238 North Renmin Road, Zhoushan, Zhejiang, 316000, China
Phone: +86 15958076211
E-mail: shibixiao1982@sohu.com

Ying Zhang, RN
NO.238 North Renmin Road, Zhoushan, Zhejiang, 316000, China
Phone: +86 13857209383
E-mail: ZZL9617@163.com

Hong Guo, RN
NO.238 North Renmin Road, Zhoushan, Zhejiang, 316000, China
Phone: +86 13675805879
E-mail: n2623@126.com

<u>Shaoxing Maternity and Child Health Care Hospital</u>

Limin Chen, RN
No.305 East Street, Shaoxing, Zhejiang, 312000, China
Phone: +86 13735377737
E-mail: cnsxclm@126.com

Xiujuan Sun, RN
No.305 East Street, Shaoxing, Zhejiang, 312000, China
Phone: +86 13757588040
E-mail: sunxiujuan0151@163.com

Ruoya Wu, RN
No.305 East Street, Shaoxing, Zhejiang, 312000, China
Phone: +86 13758596789
E-mail: 13605855788@139.com

<u>The Women & Children Hospital of Dongyang</u>

Lanzhong Guo, BS
No.40 Wuning Road, Dongyang, Zhejiang, 322100, China
Phone: +86 15967913318
E-mail: zyyglz@163.com

Xiaojiao Ye, BS
No.40 Wuning Road, Dongyang, Zhejiang, 322100, China
Phone: +86 13858968881
E-mail: yxj658881@126.com

Shiqing Lu, RN
No.40 Wuning Road, Dongyang, Zhejiang, 322100, China
Phone: +86 13625894795
E-mail: 13625894795@163.com

Ruian People's Hospital

Yuqing Cai, RN
No.108 Wansong road, Ruian, Zhejiang, 325200, China
Phone: +86 13587493618
E-mail: caiyuqing663618@163.com

Jian Zhang, RN
No.108 Wansong road, Ruian, Zhejiang, 325200, China
Phone: +86 13968936088
E-mail: zhangjian20210819@163.com

Shanhu Lin, RN
No.108 Wansong road, Ruian, Zhejiang, 325200, China
Phone: +86 15068437615
E-mail: linshanhu1988@163.com

Wenling Maternity and Child Health Care Hospital

Enli Wang, RN
No.102 Xiabao Road, Wenling, Zhejiang, 317500, China
Phone: +86 13736658870
E-mail: wangenli666@163.com

Junfei Lin, RN
No.102 Xiabao Road, Wenling, Zhejiang, 317500, China
Phone: +86 13758699860
E-mail: zjwzjhh@sohu.com

Qiaosu Chen, RN
No.102 Xiabao Road, Wenling, Zhejiang, 317500, China
Phone: +86 13906860678
E-mail: wlfbyhlb@163.com

Cixi Maternity and Child Health Care Hospital
Leiyin Cen, RN
NO.1288 Erzaotan Road, Cixi, Zhejiang, 315300, China
Phone: +86 13600610982
E-mail: ceenleiyin@126.com

Linping Jiang, RN
NO.1288 Erzaotan Road, Cixi, Zhejiang, 315300, China
Phone: +86 13968255255
E-mail: c1137959993@163.com

Linlin Feng, RN
NO.1288 Erzaotan Road, Cixi, Zhejiang, 315300, China
Phone: +86 15824573879
E-mail: fenglinlin_123@163.com

Xianju People's Hospital
Aihua Zhang, BS
No.53 Beidong Road, Xianju, Zhejiang, 317300, China
Phone: +86 13586203801
E-mail: zhangah317@xjrmyy.com.cn

Chunping Zhang, RN
No.53 Beidong Road, Xianju, Zhejiang, 317300, China
Phone: +86 13586202747
E-mail: zhangcp330@xjrmyy.com.cn

2 Study Design

2.1 Study Overview

The objective of this study was to assess the effects of acupoint hot compress during early puerperium on parturients after a vaginal delivery.

2.2 Background

Early puerperium is the period of adjustment after delivery, which extends until the first week postpartum.[1-3] It is recommended that mothers and newborns should receive care within 24 hours after birth as recommended by the World Health Organization guidelines for postnatal care, and this includes the assessment of micturition, urinary incontinence and bowel function, healing of any perineal wounds, fatigue, pain, uterine tenderness and lochia, and improving the breastfeeding progress and emotional well-being.[4] These issues can interfere with a woman's ability to care for herself and her infant.[5,6] Moreover, maternal self-care needs, already in the first few days after delivery, go beyond the mother's

physical health as it extends to the mother's emotional well-being as well.[7]

Hot compresses have proved to be an effective complementary treatment for myofascial pain syndrome in the upper trapezius muscle,[8] and they have exhibited an extraordinary capability for the pressure pain threshold and in improving the quality of life.[9] Warm compress bistage intervention was found to significantly reduce the intensity of pain the day after delivery.[10] Acupoint hot compress, with a combination of acupoints and natural physical agent heat, is more acceptable, both physically and mentally, for puerperal patients and their families, due to its noninvasive feature. However, the effects of acupoint hot compress for patients during the early puerperium remain uncertain due to a lack of evidence-based support from clinical trials.

2.3 Materials and methods

2.3.1 Objectives

The trial is designed to evaluate whether acupoint hot compress involving the abdominal, lumbosacral and plantar regions could reduce the incidence of postpartum urinary retention, relieve postpartum uterine contraction pain, prevent emotional disorders, and promote lactation.

2.3.2 Trial registration

The final protocol version is 2.1, dated 25 August 2020. The protocol was approved by the Ethics Committee of Women's Hospital, School of Medicine, Zhejiang University with the Approval No. IRB-20200223-R on 7 September 2020. The trial was registered at World Health Organization International Clinical Trial Registration Platform, Chinese Clinical Trial Registry (Chictr) with the registration number ChiCTR2000038417. Registered 22 September 2020, http://www.chictr.org.cn/showproj.aspx?proj=57108.

2.3.3 Trial design

The study is a prospective, multi-center, and randomized controlled clinical trial, which will be conducted in China. All enrolled subjects will be comprehensively briefed on the study, including the purpose, procedure, and possible risks of the study. Informed consent will be obtained from all participants before the start of this study. The demographic data, information about pregnancy, delivery, and newborns will be recorded as baseline data. When the protocol need to be modified, the implementation will be started only after the corresponding revised parts approved by the ethics committee, and all research centers, investigators and the funder are notified at the same time. The study will follow the Code of Ethics of the World Medical Association (Declaration of Helsinki). This protocol is compliant with the Standard Protocol Items: Recommendations for Interventional Trials (SPIRIT)[11] statement (Figure 1) and the Consolidated Standards of Reporting Trials (CONSORT) [12] guidelines (Figure 2).

	STUDY PERIOD								
	Enrolment	Allocation	Post-allocation			Follow-up			
**TIMEPOINT (after delivery)**	**$-t_1$**	**0**	**t_1** Within 30 minutes	**t_2** 24 hours	**t_3** 48 hours	**t_1** 6 ± 0.5 hours	**t_2** 28 ± 0.5 hours	**t_3** 52 ± 0.5 hours	**t_4** 76 ± 0.5 hours
**ENROLMENT**									
Eligibility screen	X								
Informed consent	X								
Randomization	X								
Allocation		X							
**INTERVENTIONS**									
Routine postpartum care & Acupoint hot compress			X	X	X				
Routine postpartum care			X	X	X				
**ASSESSMENTS**									
Postpartum urinary retention						X			
Postpartum uterine contraction pain (VAS)						X	X	X	X
Emotion symptoms(EPDS)									X
Lactation							X	X	X

Figure 1. The schedule of enrolment, interventions, and assessments

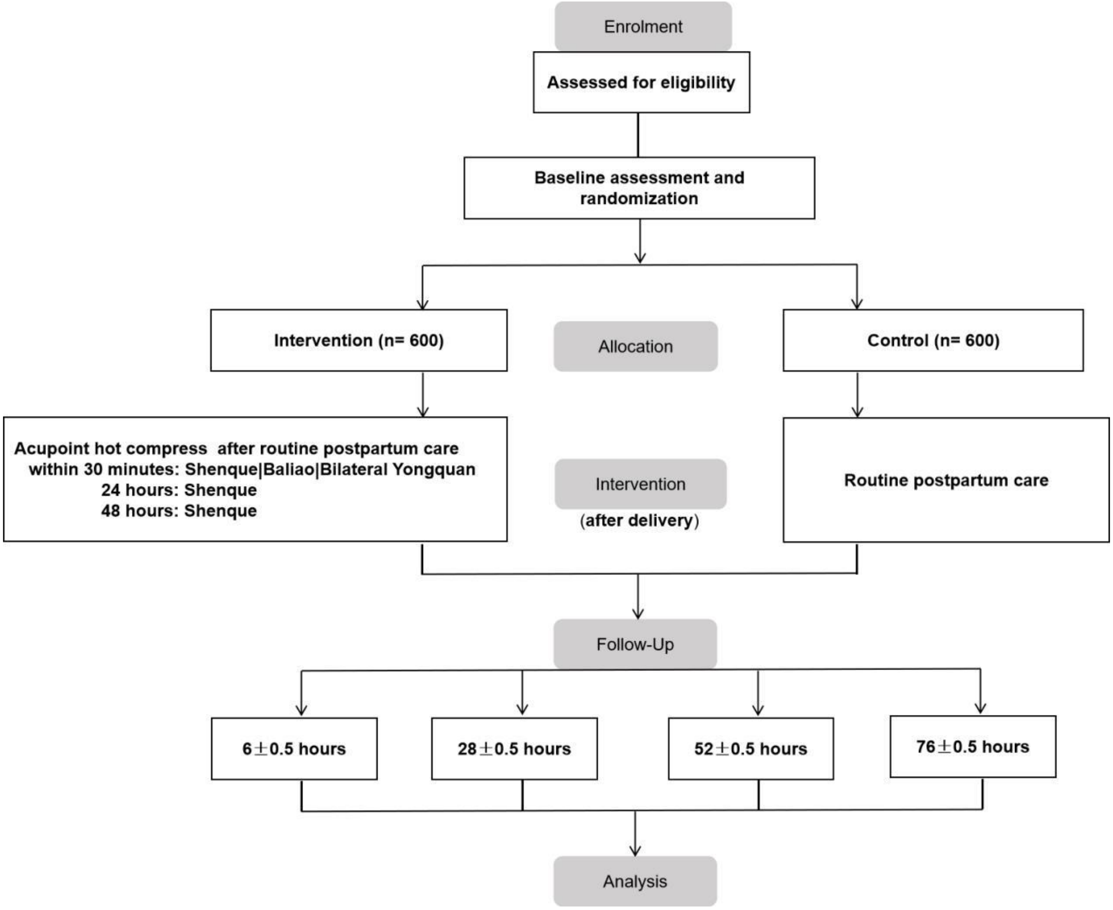

Figure 2. Trial flow chart

2.3.4 Recruitment

The study will be conducted in 12 hospitals in China: Women's Hospital, School of Medicine, Zhejiang University; Tongde Hospital of Zhejiang Province; Yiwu Maternity and Child Health Care Hospital; Jiaxing Maternity and Child Health Care Hospital; Zhejiang Xiaoshan Hospital; Zhoushan Women and Children Hospital; Shaoxing Maternity and Child Health Care Hospital; The Women & Children Hospital of Dongyang; Ruian People's Hospital; Cixi Maternity and Child Health Care Hospital; Wenling Maternity and Child Health Care Hospital and Xianju People's Hospital. Posters will be placed in each institution's bulletin board to recruit subjects. No specific culture, race, or socio-economic group will be targeted or restricted from the recruitment.

2.3.5 Participants

2.3.5.1 Inclusion criteria

(1) Age is more than 18 years;
(2) Nulliparous women with a singleton pregnancy;
(3) Gestational age of delivery is 37 - 42 weeks;
(4) Parturient undergoing vaginal delivery;
(5) Breastfeeding;
(6) Provision of the signed informed consent.

2.3.5.2 Exclusion criteria

(1) Termination of pregnancy due to fetal malformation or stillbirth;
(2) Family history of mental illness or tumor;
(3) Mental and psychological diseases, traumatic events, or communication barriers;

(4) Pre-pregnancy central nervous system diseases, internal and surgical diseases, breast dysplasia, urogenital related diseases, infectious diseases, and long-term drug treatment;

(5) Skin damage, ulceration, sensory disorders, acute closed injury, suppurative infection, acute inflammation and other infectious diseases, skin diseases, severe diabetes, high fever and allergy to product materials;

(6) Incomplete provision of information.

2.3.5.3 Subject withdrawal

On request, the enrolled subjects can withdraw from the clinical trial at any stage of the study.

2.3.5.4 Subject drop-out and discontinuation

(1) Implementation of rescue due to postpartum hemorrhage, eclampsia, or other obstetric conditions;

(2) Change of vaginal delivery to cesarean section;

(3) Infection during delivery;

(4) Indwelling catheter and the need to use diuretics due to disease within 6 hours after delivery;

(5) Degree III laceration above the perineal fissure;

(6) Neonatal transfer after birth;

(7) The investigator can terminate the subject from continuing the clinical trial at any stage, if the investigator deems that the risks of continuing the clinical trial outweigh the benefits;

(8) Subject's poor compliance to this trial.

2.4 Sample size calculation

This study will use a superiority design. According to the pilot study, the incidence rate of urinary retention was 7.7% in the intervention group and 25.7% in the control group. Assuming a 1 : 1 ratio of intervention to control group and using a one-sided test, a significance level (α) of 0.025 and a power ($1-\beta$) of 80% and a possible drop-out rate of 20%, the estimated sample size is 600 cases in each group with 50 cases at each center in each group. Intervention and control groups would require a total sample size of 1200 cases.

2.5 Randomization and blinding

Eligible subjects will be randomly assigned to the intervention group or control group in a ratio of 1:1. The randomization number sequence will be generated with R software by an independent statistician. Randomization number sequence will be stored by a non-involved investigator. The design is open label, but the outcome assessors and statisticians who are blinded to group allocation will be responsible for collecting and analyzing the data, respectively.

2.6 Interventions

2.6.1 Control group

The participants in the control group received routine postpartum care, including the observation of their vital signs (blood pressure, heart rate, respiratory rate, temperature and oxygen saturation of blood), the monitoring the amount of vaginal bleeding and discharge of lochia, the palpation of their fundus, cleansing of their vulva and an early interaction with their newborns.[13]

2.6.2 Intervention group

In addition to routine postpartum care, the participants in the intervention group were administered a 4-hour acupoint hot compress with a constant temperature of 45±2°C within 30 minutes after delivery (Intervention I), 24 hours after delivery (Intervention II), and 48 hours after delivery (Intervention III).

As seen in Figure 3, for Intervention I, two hot cores (Model A) were administered on Shenque (RN8, in the center of the umbilicus) and Baliao (BL31-34, in the region of the sacrum, between the posterior-superior iliac spine and the posterior midline, in the 1st, 2nd, 3rd and 4th posterior sacral foramen, from the top to the bottom are Shangliao, Ciliao, Zhongliao, and Xialiao). Two hot cores (Model B) were bilaterally administered in Yongquan (KI1, on the sole, in the depression when the foot was in plantar flexion, and in the anterior depression when the foot was flexed, approximately at the junction of the anterior 1/3 and posterior 2/3 of the sole). During Interventions II and III, only one hot core (Model A) was administered on Shenque (RN8). The selection of acupoints is based on the clinical experiences of our hospital and the consultation with 10 experts in Chinese medicine. The location of the involved acupoints is described as per the Nomenclature and Location of Acupuncture Points (National Standard of People’s Republic of China, 2006 [GB/T 12346-2006]).

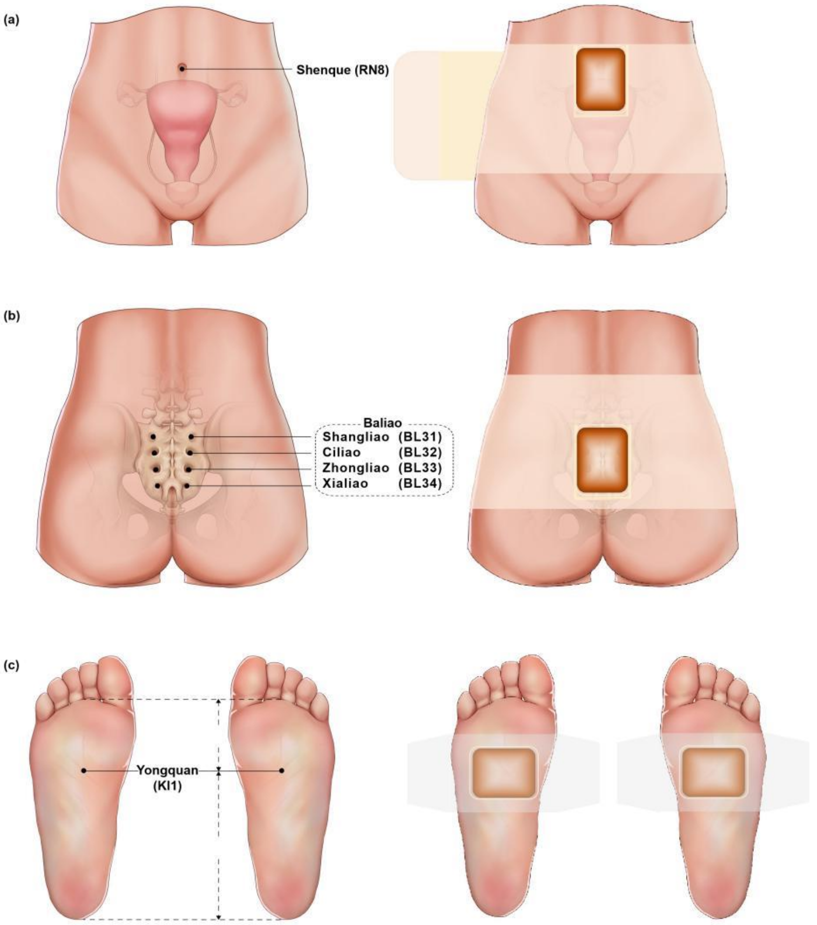

Figure 3. Acupoints

(a) Shenque (RN8), in the center of the umbilicus;

(b) Baliao(BL31-34), in the region of the sacrum, between the posterio-superior iliac spine and the posterior midline, in the 1st, 2nd, 3rd and 4th posterior sacral foramen, from top to bottom are Shangliao, Ciliao, Zhongliao, and Xialiao;

(c) Yongquan (KI1), on the sole, in the depression when the foot is in plantar flexion, and in the anterior depression when the foot is flexed, approximately at the junction of the anterior 1/3 and posterior 2/3 of the sole.

The hot compress was applied using a licensed Class II medical device, Hu-Chao-Nuan-Gong-Bao (produced by Jiangxi Shenghe Industrial Development Co., Ltd., Nanchang, China, with a license No. 20192090292). Four hot cores of Model A (11.5×8 cm with an entire patch of 13×10 cm) and another two hot cores of Model B (8×7 cm with an entire patch of 16×9 cm) were included in the licensed device. The material layer of a hot core consists of iron powder, water, activated carbon and inorganic salts, which are put into a sealed inner bag with a specific proportion. A specific water vapor transmission rate and a specific oxygen transmission rate of the air-permeable layer can keep a constant temperature (Figure 4).

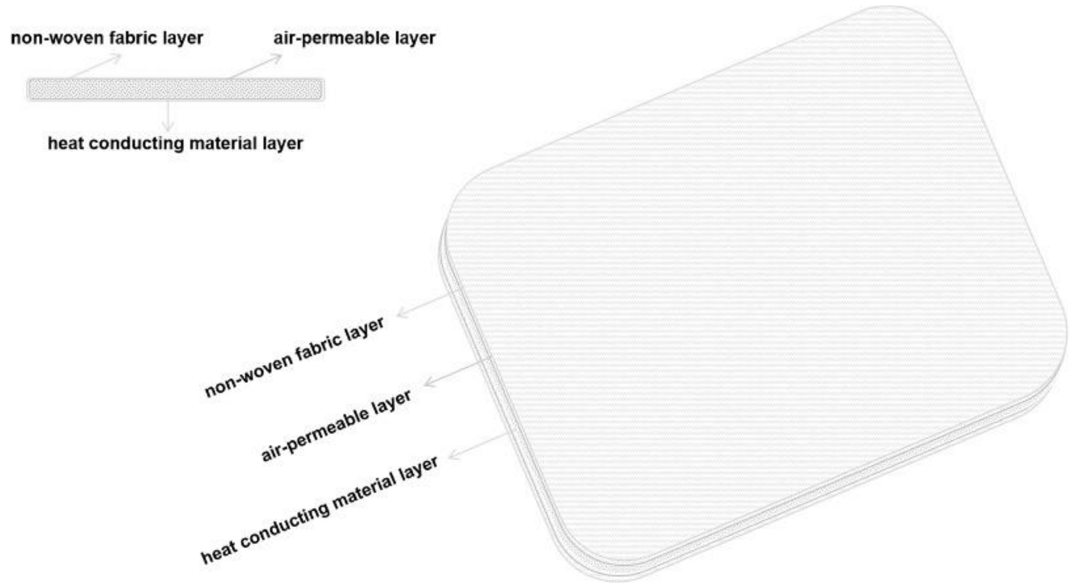

Figure 4. Structure diagram of hot core: From the outside to the inside are the non-woven fabric layer, air-permeable layer and heat conducting material layer

2.7 Baseline data

Baseline information includes: Age; BMI(Prepregnancy); Race; ABO blood groups; Rh blood types; Place of residence; Educational level; Occupation category; History of tobacco consumption; History of alcohol intake; Insurance; Age of menarche; Menstrual cycle; Menstrual phase; Dysmenorrhea; Marital status; Couple relationship; Mode of conception; Prenatal care; Pregnancy complications; Fetus and appendages; Gestational age of delivery; BMI(Predelivery); Sleep duration per day; Trouble falling asleep; Appetite; Little interest or pleasure in doing things; Feeling down, depressed, or hopeless; Feeling nervous, anxious or on edge; Not being able to stop or control worrying; Labor induction; Augmentation by oxytocin; Rupture of membranes; Spinal analgesia for labor pain; Duration of labor stages; Perineum conditions; Assisted vaginal delivery; Placenta delivery method; Completeness of placenta; Fetal membranes; Curettage; Uterotonics drugs; Bowel movement during labor; Newborn weight; Newborn length; Apgar score; Time between birth and early sucking of newborns; Knowledge of breastfeeding; Postpartum diet within 28 hours after delivery; Postpartum diet during 28-52 hours after delivery; Postpartum diet during 52-76 hours after delivery; Postpartum appetite within 28 hours after delivery; Postpartum appetite during 28-52 hours after delivery; Postpartum appetite during 52-76 hours after delivery.

2.8 Outcomes measures

2.8.1 Primary outcome

The time point of the first urination after delivery and whether an indwelling catheter was used within 72 hours after delivery will be recorded. In the present trial, postpartum urinary retention is defined as the first urination occurring more than 6.5 hours after delivery and/or if an indwelling catheter is used within 72 hours after delivery.[4,14-16]

2.8.2 Secondary outcomes

Postpartum uterine contraction pain: The visual analogue scale (VAS) will be used to evaluate the postpartum uterine contraction pain intensity: Using a horizontal ruler of 100 mm, “0” representing no pain and “100” representing unbearable pain. The pain VAS intensity will be respectively measured at 6 ± 0.5 hours, 28 ± 0.5 hours, 52 ± 0.5 hours, and 76 ± 0.5 hours after delivery.[17]

Emotion symptoms: The Edinburgh Postnatal Depression Scale (EPDS) will be used for screening the depressive symptoms at 76 ± 0.5 hours after delivery. It contains 10-item: pessimism, lack of interest, self-blame, worry, fear, impaired ability, sleep disorder, sadness, tearfulness, self-injury/suicidal ideation, each of which is scored from 0 to 3.[6]

Lactation: The lactation initiation time will be recorded, and the breastfeeding milk volume, feeding mood and times, and the newborn weight will be measured at 28±0.5, 52±0.5, and 76±0.5 hours after delivery, respectively. The breastfeeding milk volume is measured as “-” to “++++”: no milk outflow during finger extrusion(-); milk occasionally flows out during finger extrusion, which cannot meet the needs of the infants (+); milk flows out slowly when squeezed by fingers but does meet the needs of infants (+ +); milk continuously flows out during finger extrusion, which can meet the needs of infants (+ + +); there is milk flowing out in a jet shape when the nipple is squeezed by the fingers, which can meet the needs of infants (+ + + +).[18,19]

2.9 Safety Evaluation

The medical device for hot compress used in the present study is licensed by Medical Products Administration (Jiangxi Province, China) with a Licensed Number 20192090292. No adverse events (AEs) have been reported. In the present study, the safety evaluation will be comprehensively conducted and followed. In the study, the participants with sensory disorders have been excluded. Prior to obtaining consent from the participants, the potential AEs have been explained to the participants in detail. AEs will be monitored and recorded in detail throughout the whole study period.

3. Informed Consent

Notification Page

Dear:

You will be invited to participate in a prospective, multi-center, and randomized controlled clinical trial on the effects of acupoint hot compress on early puerperal rehabilitation of parturients with vaginal delivery. Before you decide whether to participate in this study, please read the following contents as carefully as possible. It can help you understand why it is conducted, the procedures and duration of the study, benefits, risks and discomforts. You can feel free to ask the investigator for explanations to help you make your decision.

I. Study Background

Puerperal diseases negatively affect the physical and mental health of parturients and the growth and development of infants, and even the development of society. In face of a large reproductive-age population, it is particularly important to evaluate and prevent early puerperium urinary retention, uterine contraction pain, emotional and lactation disorders.

Traditional Chinese medicine (TCM) is one of the oldest healing systems. TCM has obvious advantages in early rehabilitation of puerperal patients with its unique philosophical and theoretical system, rich clinical practice experience and the mode of combining life, society, environment and psychology. Acupoint hot compress, as a nonpharmacologic therapy, is an organic combination of external treatment and physical therapy of TCM. Acupoints or points, are the specific site where Qi and Zang-fu organs and meridians are transported to the body surface. They possess the functions of receiving stimulation and preventing and curing diseases. Hot compress, a kind of thermal therapy, acts on the body through physiological regulatory mechanisms such as nerves, body fluids, and endocrine to improve blood and lymphatic circulation, promote tissue metabolism, reduce muscle tension, relieve spasm as well as analgesia, and prevent and treat diseases with a safe, effective, simple and cheap manner.

In this study, with applications of Shenque acupoint of Ren meridian, Baliao acupoint of Bladder Meridian of Foot-Taiyang, Yongquan acupoint of Kidney Meridian of Foot-Shaoyin, acupoints combined with natural physical agent heat can provide a simple and clean intervention for reduction of the incidence of postpartum urinary retention, relief of postpartum uterine pain, prevention of emotional disorders, promotion of lactation, and to ensure the health of women throughout the life.

II. Purpose of Study

To access the effects of early puerperal acupoint hot compress in reducing the incidence of postpartum urinary retention, relieving postpartum uterine pain, promoting lactation, and preventing mood disorders, which could provide a scientific basis for the application of non-drug therapy in puerperal rehabilitation.

III. Study methods and contents

We will give a random number to each participant and establish a file. Based on the randomization number, you will be randomized to one of the two groups. After routine postpartum care, the intervention group will be given acupoint hot compress at a constant temperature of 45±2°C within 30 minutes after vaginal delivery, 24 hours after delivery, and 48 hours after delivery, with 4 hours each time, and the control group will be given routine postpartum care only.

Acupoint hot compress will be applied with a licensed medical device Hu-Chao-Nuan-Gong-Bao with produced by Jiangxi Shenghe Industrial Development Co., Ltd., Nanchang, China.

We will assess the postpartum urinary retention at 6±0.5 hours after delivery, postpartum uterine contraction pain at 6±0.5 hours, 28±0.5 hours, 52±0.5 hours, and 76±0.5 hours after delivery, lactation at 28±0.5 hours, 52±0.5 hours, and 76±0.5 hours after delivery, and postpartum depressive symptoms at 76 ± 0.5 hours after delivery.

IV. Estimated Duration of Study

From 1 August 2020 to 31 July 31 2023

V. Expected number of subjects

The intervention group and control group require a total of 1200 cases, and there are 600 cases in each group .

VI. Inclusion and exclusion criteria

Inclusion criteria: (1) Age is more than 18 years; (2) Nulliparous women with a singleton pregnancy; (3) Gestational age of delivery is 37-42 weeks; (4) Parturient undergoing vaginal delivery; (5) Breastfeeding;

(6) Provision of the signed informed consent.

Exclusion criteria: (1) Termination of pregnancy due to fetal malformation or stillbirth; (2) Family history of mental illness or tumor; (3) Mental and psychological diseases, traumatic events, or communication barriers; (4) Pre-pregnancy central nervous system diseases, internal and surgical diseases, breast dysplasia, urogenital related diseases, infectious diseases, and long-term drug treatment; (5) Skin damage, ulceration, sensory disorders, acute closed injury, suppurative infection, acute inflammation and other infectious diseases, skin diseases, severe diabetes, high fever and allergy to product materials; (6) Incomplete provision of information.

Subject withdrawal: On request, the enrolled subjects can withdraw from the clinical trial at any stage of the study.

Subject drop-out and discontinuation: (1) Implementation of rescue due to postpartum hemorrhage, eclampsia, or other obstetric conditions; (2) Change of vaginal delivery to cesarean section; (3) Infection during delivery; (4) Indwelling catheter and the need to use diuretics due to disease within 6 hours after delivery; (5) Degree III laceration above the perineal fissure; (6) Neonatal transfer after birth; (7) The investigator can terminate the subject from continuing the clinical trial at any stage, if the investigator deems that the risks of continuing the clinical trial outweigh the benefits; (8) Subject's poor compliance to this trial.

VII. Risks and Benefits to Subjects

Risks: In the previous studies, there have been no adverse events of this medical device since post-marketing. Acupoint hot compress and the follow-up assessments during the study may take up some of your time. The use of acupoint hot compress (Hu-Chao-Nuan-Gong-Bao) in the intervention group may not achieve the expected efficacy. The allergy and burns are extremely rare.

Prevention of risks: If you are randomly assigned to the intervention group and if adverse event occurs during the application of the medical device, you can call the investigator at any time. The investigator will fully explain and give effective guidance to you. During the study, we will strictly control the inclusion and exclusion criteria.

Benefits: Those randomized to the intervention group during the study may intervene to reduce the incidence of postpartum urinary retention, relieve postpartum uterine pain, promote lactation, prevent mood disorders, which has positive effects on puerperal health. Although there is evidence to indicate a satisfactory response, this does not guarantee that it will work for you. During the study, we will inform you in a timely manner if we learn of any information that may affect your continued participation in the study.

VIII. Costs and Compensation

Cost: The cost of acupoint hot compress in this study will be covered by the investigator, and the cost of routine diagnosis and other treatment items will be self-paid. We will make effort to prevent and treat possible adverse events due to this study. If an adverse events occurs, whether or not related to this study, you will receive timely treatment. If confirmed that it is related to this study, we will provide the cost of diagnosis, treatment and the corresponding compensation for the study-related damage in accordance with the provisions of relevant laws or guidelines in China.

Compensation: After all the follow-up assessments are completed, we will pay RMB 100 Yuan per subject.

IX. Confidentiality

Records about your identity are kept confidential and your name will not appear on case report forms(CRFs), any relevant study reports and public publications. Your medical data will be used for

scientific research only and will not be used for any commercial purposes. The investigator, sponsor representative, monitors, ethics committee and regulatory authorities will be allowed access to your medical records. We will make effort to protect the privacy of your personal medical data to the extent permitted by law.

You have the right to learn about yourself at any time during the study.

X. Voluntary principle

Whether to participate in the study depends entirely on your wishes. You may refuse to participate in the study or withdraw from the study at any time during the study. This will not affect your medical treatment or other benefits.

The doctor or investigator may discontinue your participation in the study at any time during the study if you require other treatment, do not follow the study plan, or have an injury related to the study or for any other reason.

If you withdraw from the study for any reason, you may be asked about your experiences of using the medical device. You may also be asked to have relevant laboratory tests and physical examinations if your doctor thinks it is necessary.

XI. Subject Responsibilities

①If you are a qualified participant, you may voluntarily participate in the study and sign the informed consent form. If you are unwilling to participate in this study, other appropriate treatment will be given according to your condition and wishes.

②If you are willing to participate in the study, you may be assigned to different groups, and you need to cooperate with the investigator for examination and follow-up.

③You will have to undergo the evaluations and observations according to the follow-up time agreed by the investigator and you, and please fill in your relevant records in a timely and objective manner.

XII. How to get more information

During the study, if you have any questions or do not understand anything about the study, you can ask the investigator at any time. We will inform you any important new information that may affect your willingness to continue to participate in the study in a timely manner.

For more questions, please contact the Ethics Committee, Tel.:+86 571 89992355.

XIII.Other things the patient should know

It is up to you to decide whether or not to take part.

Before make your decision, ask your doctor or investigator as many questions as possible until you fully understand the study.

Thank you for reading the above information. If you decide to take part in this study, please tell your doctor or investigator, who will arrange everything related to the study for you.

Signature Page

Title:Effects of acupoint hot compress during early puerperium on parturients after a vaginal delivery: a randomized clinical trial

Project leader: Fan Qu

Sponsor: Women’s Hospital, School of Medicine, Zhejiang University

Approved No. of ethic committee: IRB-20200223-R

Consent Statement

I have carefully read the above introduction on this study, and the investigator has explained the characteristics and the possible items of this study to me in detail and has answered the relevant

questions.
After fully understanding all the information for subjects and the risks and benefits of participating in the trial, I have sufficient time to think about it and I will voluntarily participate in the trial, fully cooperate with the investigators, truthfully and objectively provide the investigators with the health status and related conditions before participating in the study.
I understand that I can consult investigator for more information at any time, and I can choose not to participate in this study, or quit at any time after informing the researcher without any discrimination or reprisals, and my medical treatment and rights will not be affected.
I am also aware that if I withdraw from the study halfway, especially due to the use of the medical device. To inform the investigator of the changes of my conditions and complete the corresponding physical examinations and chemical examinations, will be very beneficial to the whole study. If I need to take any other medication due to changes of my conditions, I will ask the investigator for advice in advance or tell the investigator truthfully afterwards. The investigator may stop me from continuing in the study if I do not follow the study plan or if I have an injury or any other reason related to the study.
I understand that I will receive a signed copy of the informed consent.
Finally, I fully agree to participate this study and to make effort to follow the instructions.

Signature of Subject:
Signature of legal representative (if necessary):
(Relationship with the subject):
Witnessed by: (if necessary):
Tel.:
Date: D/M/Y
Signature of Investigator:
Tel.:
Date: D/M/Y

Contact us: Yuhang Zhu
Tel.: +86 15267047921

Ethics Committee of Women’s Hospital, School of Medicine, Zhejiang University
Tel.:+86 571 89992355

4. Risks

In previous studies and clinical practice, acupoint hot compress used by Hu-Chao-Nuan-Gong-Bao has not been reported to have any adverse event.

If the subject experiences any adverse event, she can call the researcher at any time, and the researcher will fully explain and provide effective guidance. If an adverse event occurs, no matter it is related to this study or not, the subject will be treated in time. After confirmation by relevant experts from medical institutions and centers, the treatment costs will be covered by the clinical study sponsor and appropriate economic compensation will be given according to relevant national regulations.

5. Benefits

During the study, allocation to acupoint hot compress intervention group may reduce the incidence

of postpartum urinary retention, relieve postpartum uterine contraction pain, prevent emotional disorders, and promote lactation, which has a positive effect on puerperal health. Although there are evidences to suggest a satisfactory response, this does not guarantee a positive response for each subject. The intervention used in this study is not the only choice. If it is ineffective for the subject's condition, the subject can ask the physician for alternative treatment. During the study, if we learn of any information that may affect the continued participation of parturients in the study, the subject will be informed in time.

6. Data Management

All the investigators will receive standard training and operate strictly according to the standard procedure. Double data entry will be applied to ensure the accuracy. A data monitoring committee will be set up before the first enrollment of participants. The original data will be collected and recorded in case report forms (CRFs). Each participant and the investigator will sign and record the date on CRFs, which will be maintained in a secure location. All the data and CRFs during the study are available from the corresponding author on reasonable requests. After publication of the study, all the relevant data will be stored for 3 years in Women’s Hospital, School of Medicine, Zhejiang University.

7. Statistical analysis

The following is an overview of the statistical considerations. Details of the pre-specified statistical analyses can be found in Statistical Analysis Plan (SAP).

Statistical analysis will be performed by independent analysts by using R (version 4.1.0; R Development Core Team) software. All the analyses will be based on an intention-to-treat principle. Continuous data will be represented by the average, standard deviation, median, minimum and maximum value; categorical data will be represented by the number and percentages. Continuous data will be analyzed using the t test or one-way ANOVA; Wilcoxon-Mann-Whitney test will be used when necessary. Categorical data will be analyzed using the chisquare test or Fisher’s exact test. The logistic regression model or linear regression model and linear mixed model will also be used to find the relationship between independent variables. The subgroup relative risk (RR) and the corresponding 95% confidence intervals (CIs) are calculated together with interaction P values. $P < 0.05$ will be considered statistically significant.

8. Privacy

If the subject decides to participate in this study, the data of the subject recorded throughout the study are only used for scientific research purpose and not for any news or commercial ones. The personal data of the subjects involved will be kept strictly confidential. When the study are published, none of the personal privacy such as the name and contact information of the subjects will be identified. After the study is published, all the study materials will be preserved in Women’s Hospital School of Medicine, Zhejiang University for verification of study implementation required by national regulatory authorities, scientific research management authorities and Ethics Committee.

9. Funding

This study is supported by Association for Maternal and Child Health, Zhejiang Province, China

(ZY2020-01). The funder is responsible for the administrative and financial management of the study, and not involved in the design of the study, nor in the collection, analysis, and interpretation of data or in writing the manuscript.

10. Reference

(1) ACOG Committee Opinion No. 736: Optimizing Postpartum Care. *Obstet Gynecol*. 2018;131(5):e140-e150.

(2) Kristoschek JH, Moreira de Sá RA, Silva FCD, Vellarde GC. Ultrasonographic Evaluation of Uterine Involution in the Early Puerperium. *Rev Bras Ginecol Obstet*. 2017;39(4):149-154.

(3) Xue FX, Ma YY. Obstetrics and Gynecology. In Li XT. (ed.) Puerperium. Beijing: Tsinghua University Press. 2017:621-626.

(4) WHO recommendations on maternal health: guidelines approved by the WHO Guidelines Review Committee. World Health Organization. 2017:16-19.

(5) ACOG Committee Opinion No. 742: Postpartum Pain Management. *Obstet Gynecol*. 2018;132(1):e35-e43.

(6) Meltzer-Brody S, Howard LM, Bergink V, et al. Postpartum psychiatric disorders. *Nat Rev Dis Primers*. 2018;4:18022.

(7) Lambermon F, Vandenbussche F, Dedding C, van Duijnhoven N. Maternal self-care in the early postpartum period: An integrative review. *Midwifery*. 2020;90:102799.

(8) Boonruab J, Damjuti W, Niempoog S, Pattaraarchachai J. Effectiveness of hot herbal compress versus topical diclofenac in treating patients with myofascial pain syndrome. *J Tradit Complement Med*. 2019;9(2):163-167.

(9) Boonruab J, Nimpitakpong N, Damjuti W. The Distinction of Hot Herbal Compress, Hot Compress, and Topical Diclofenac as Myofascial Pain Syndrome Treatment. *J Evid Based Integr Med*. 2018;23:2156587217753451.

(10) Akbarzadeh M, Vaziri F, Farahmand M, Masoudi Z, Amooee S, Zare N. The Effect of Warm Compress Bistage Intervention on the Rate of Episiotomy, Perineal Trauma, and Postpartum Pain Intensity in Primiparous Women with Delayed Valsalva Maneuver Referring to the Selected Hospitals of Shiraz University of Medical Sciences in 2012-2013. *Adv Skin Wound Care*. 2016;29(2):79-84.

(11) Chan AW, Tetzlaff JM, Altman DG, et al. SPIRIT 2013 statement: defining standard protocol items for clinical trials. *Ann Intern Med*. 2013;158(3):200-207.

(12) Moher D, Hopewell S, Schulz KF, et al. CONSORT 2010 explanation and elaboration: updated guidelines for reporting parallel group randomised trials. *BMJ (Clinical research ed)*. 2010;340:c869.

(13) Obstetrics Subgroup, Chinese Society of Obstetrics and Gynecology, Chinese Medical Association; Chinese Society of Perinatal Medicine, Chinese Medical Association. Guideline of normal birth. *Chin J Obstet Gynecol*. 2020;55(6):361-370. [In Chinese]

(14) Yip SK, Sahota D, Pang MW, Day L. Postpartum urinary retention. *Obstet Gynecol*. 2005;106(3):602-606.

(15) Cavkaytar S, Kokanalı MK, Baylas A, Topçu HO, Laleli B, Taşçı Y. Postpartum urinary retention after vaginal delivery: Assessment of risk factors in a case-control study. *J Turk Ger Gynecol Assoc*. 2014;15(3):140-143.

(16) Gu SF, Li MQ, Li Y, Chen M, Han LL. Acupuncture at Ciliao (BL 32) for prevention of postpartum urinary retention in elderly parturient women: a randomized controlled trial. *Zhongguo Zhen Jiu*. 2020; 40: 611–614. [In Chinese]

(17) Olsén MF, Elden H, Janson ED, Lilja H, Stener-Victorin E. A comparison of high- versus low-intensity, high-frequency transcutaneous electric nerve stimulation for painful postpartum uterine contractions. *Acta Obstet Gynecol Scand*. 2007;86(3):310-314.
(18) Kellams A, Harrel C, Omage S, Gregory C, Rosen-Carole C. ABM Clinical Protocol #3: Supplementary Feedings in the Healthy Term Breastfed Neonate, Revised 2017. *Breastfeed Med*. 2017;12:188-198.
(19) Hartmann P, Cregan M. Lactogenesis and the effects of insulin-dependent diabetes mellitus and prematurity. *J Nutr*. 2001;131(11):3016s-3020s.

11. Update on the Published Protocol

As compared to the published protocol (Yuhang Zhu, Aihua Zhang, Chang Liu, Nicola Robinson, Myeong Soo Lee, Xiaoyang Mio Hu, Hye Won Lee, Rong Zhang, Fan Qu*. Effectiveness of acupoint hot compress on early puerperal rehabilitation of parturients after natural childbirth: study protocol for a prospective, multi-center, randomized controlled clinical trial. Clinical and Experimental Obstetrics & Gynecology.2021;48(6):1350-1357), the present study protocol has made several amendments.

(1) The medical provision of two centers changed, which has limited the number of the available participants with inclusion criteria of the study, and these two centers were not included in the trial.
(2) The analysis used in the present trial is referred to as an “per protocol” analysis.
(3) To detect the effects of acupoint hot compress on reducing the incidence of urinary retention more accurately, we used a broader standard to identify postpartum urinary retention.
(4) To detect the effects of acupoint hot compress on lactation, we used a detailed measurement to quantify the breastfeeding milk volume.
(5) As all the outcomes collected from 6±0.5 hours to 76±0.5 hours after delivery, the changes of maternal weight is tiny, so the weight was not included in the analysis.

Effects of acupoint hot compress during early puerperium on parturients after a vaginal delivery: study protocol for a randomized clinical trial

Statistical Analysis Plan

Final version: August 25, 2020

Trial Registration: Chinese Clinical Trial Registry(Registration number ChiCTR2000038417).

Prepared for and approved by the Data committee of the study:

Dr. Fan Qu (Chair), Women's Hospital, School of Medicine, Zhejiang University, China

Dr. Yuhang Zhu (Member), Women's Hospital, School of Medicine, Zhejiang University, China

Dr. Fangfang Wang (Member), Women's Hospital, School of Medicine, Zhejiang University, China

Dr. Jue Zhou (Member), Zhejiang Gongshang University, China

Dr. Yan Wu (Member), Women's Hospital, School of Medicine, Zhejiang University, China

Prepared by an Independent Statistician:

Yaoyao Lin (Statistician), Department of Epidemiology and Biostatistics, Zhejiang University School of Medicine, China

Mengling Tang (Statistician), Department of Epidemiology and Biostatistics, Zhejiang University School of Medicine, China

Table of Contents

1. Study Populations 26
2. Analysis Software 26
3. Missing Data 26
4. Analyses for Baseline Analysis 26
5. Analyses for the Primary Outcome 26
6. Analyses for Secondary Outcome 27
7. Subgroup analysis 27
8. Safety Analysis 27
9. Changes to the original analysis plan 27

1. Study Populations

All subjects with randomization will be included in the analysis set regardless of whether they receive any treatment. According to the intention-to-treat principle, all analysis will be based on the randomization set.

2. Analysis Software

Statistical analysis will be performed by independent analysts by R (version 4.1.0; R Development Core Team) software. All hypothesis testing will be carried out at the 5% (2-sided) significance level.

3. Missing Data

Missingness is considered to be missing at random (MAR) and multiple imputation (MI) techniques will be used for handling missing data.

4. Analyses for Baseline Analysis

Baseline information includes: Age; BMI(Prepregnancy); Race; ABO blood groups; Rh blood types; Place of residence; Educational level; Occupation category; History of tobacco consumption; History of alcohol intake; Insurance; Age of menarche; Menstrual cycle; Menstrual phase; Dysmenorrhea; Marital status; Couple relationship; Mode of conception; Prenatal care; Pregnancy complications; Fetus and appendages; Sleep duration per day; Trouble falling asleep; Appetite; Little interest or pleasure in doing things; Feeling down, depressed, or hopeless; Feeling nervous, anxious or on edge; Not being able to stop or control worrying; Labor induction; Augmentation by oxytocin; Rupture of membranes; Spinal analgesia for labor pain; Duration of labor stages; Perineum conditions; Assisted vaginal delivery; Placenta delivery method; Completeness of placenta; Fetal membranes; Curettage; Uterotonics drugs; Bowel movement during labor; Gestational age of delivery; BMI(Predelivery); Newborn weight; Newborn length; Apgar score; Time between birth and early sucking of newborns; Knowledge of breastfeeding; Postpartum diet within 28 hours after delivery; Postpartum diet during 28-52 hours after delivery; Postpartum diet during 52-76 hours after delivery; Postpartum appetite within 28 hours after delivery; Postpartum appetite during 28-52 hours after delivery; Postpartum appetite during 52-76 hours after delivery.

For continuous variables, means and standard deviations(SD) will be presented, unless the variable has a skewed distribution, in which case medians, 25th and 75th percentiles will be presented. For categorical variables, the number and percentage of participants within each category will be presented. For each variable (continuous or categorical), the number of missing values will be reported. Continuous data will be analyzed using the t test or Wilcoxon rank- sum test. Categorical data will be analyzed using the chi-square test or Fisher's exact test.

5. Analyses for the Primary Outcome

For the primary outcome, comparisons of urinary retention between intervention group and control group will be performed using logistic regression model without any adjustment; relative risk (RR) and corresponding 95% confidence intervals(CIs) will be calculated.

6. Analyses for Secondary Outcome

For the secondary outcomes, the changes of postpartum uterine contraction pain assessing with Visual Analogue Scale Score at 6±0.5, 28±0.5, 52±0.5and 76±0.5 hours after birth will be analyzed by fitting a linear mixed model using treatment, time, treatment by time interaction as a fixed effect, the comparison between the two groups at each time-point will be performed using linear regression model.

The emotion symptoms screening by the Edinburgh Postnatal Depression Scale Score will be compared using a logistic regression model.

The lactation initiation time will be compared using the Wilcoxon rank-sum test. The changes of breastfeeding milk volume, feeding mood and feeding times at 28±0.5, 52±0.5 and 76±0.5 hours after birth will be analyzed by a generalized linear mixed model. The difference of breast feeding milk volume and feeding times between two groups at each time-point will be analyzed by an ordinal logistic regression model, and feeding mood will be analyzed by a logistic regression model. The changes of newborn weight at 28±0.5, 52±0.5 and 76±0.5 hours after birth will be analyzed by fitting a linear mixed model using treatment, time, treatment by time interaction as a fixed effect, the comparison between the two groups at each time-point will be performed using a linear regression model.

7. Subgroup analysis

To consider the potential effects of labor analgesic drug use on the outcomes, a subgroup analysis will be conducted according to whether medication is used for labor analgesia or not. The subgroup RRs and 95% CIs will be calculated together with interaction P values.

8. Safety Analysis

Adverse event listings will be generated and events will be classified according to seriousness, treatment relationship and resolution. Descriptive statistics (numerical measures, frequency tables) will be used to summarize the occurrence of adverse events and serious adverse events by intervention group.

9. Changes to the original analysis plan

As compared to the initial statistical analysis plan (SAP) published in Clinical and Experimental Obstetrics & Gynecology(Yuhang Zhu, Aihua Zhang, Chang Liu, Nicola Robinson, Myeong Soo Lee, Xiaoyang Mio Hu, Hye Won Lee, Rong Zhang, Fan Qu*. Effectiveness of acupoint hot compress on early puerperal rehabilitation of parturients after natural childbirth: study protocol for a prospective, multi-center, randomized controlled clinical trial. Clinical and Experimental Obstetrics & Gynecology. 2021;48(6):1350-1357), the original analysis plan has been amended as follows:
Based on the preliminary analysis of the available data, we found spinal analgesia may have potential effects on the outcomes, a subgroup analysis was then conducted.
