# Supplementary material for: Effect of Acupoint Hot Compress on Postpartum Urinary Retention After Vaginal Delivery: A Randomized Clinical Trial
Source: JAMA Netw Open. 2022 May 23;5(5):e2213261. doi: 10.1001/jamanetworkopen.2022.13261 (PMC9127553; doi:10.1001/jamanetworkopen.2022.13261)
Supplement: Supplement 2. — eTable 1. Secondary Analysis Lactation eTable 2. Subgroup Analysis for Participants With or Without Spinal Analgesia Lactation [file jamanetwopen-e2213261-s002.pdf]

Supplemental Online Content

Zhu Y, Wang F, Zhou J, et al. Effect of acupoint hot compress on postpartum urinary retention after vaginal delivery: a randomized clinical trial. *JAMA Netw Open*. 2022;5(5):e2213261. doi:10.1001/jamanetworkopen.2022.13261

eTable 1. Secondary Analysis Lactation
eTable 2. Subgroup Analysis for Participants With or Without Spinal Analgesia Lactation

This supplemental material has been provided by the authors to give readers additional information about their work.

© 2022 Zhu Y et al. *JAMA Network Open.*

eTable 1. Secondary Analysis Lactation

**Lactation**														
Lactation initiation time[a], median (IQR), mins			367.0(82.00-1265.00)					434.5(114.00-1480.75)					NA	.18
Breastfeeding milk volume[b]	Volume		-	+	++	+++	++++	-	+	++	+++	++++		
	Time point	28.5 hours	67 (12.5)	361 (67.2)	108 (20.1)	1 (0.2)	0 (0.0)	97 (17.7)	384 (70.1)	61 (11.1)	6 (1.1)	0 (0.0)	NA	<.001
		52.5 hours	7 (1.3)	115 (21.4)	326 (60.7)	87 (16.2)	2 (0.4)	10 (1.8)	154 (28.1)	322 (58.8)	61 (11.1)	1 (0.2)	NA	.02
		76.5 hours	0 (0.0)	29 (5.4)	156 (29.1)	251 (46.7)	101 (18.8)	1 (0.2)	40 (7.3)	208 (38.0)	253 (46.2)	46 (8.4)	NA	<.001
Feeding mood[b]	Mood		Exclusive breastfeeding		Partial breastfeeding			Exclusive breastfeeding		Partial breastfeeding				
	Time point	within 28 hours after delivery	289(53.8)		248(46.2)			274(50.0)		274(50.0)			NA	.22
		during 28-52 hours after delivery	258(48.0)		279(52.0)			261(47.6)		287(52.4)			NA	.90
		during 52-76 hours after delivery	318(59.2)		219(40.8)			327(59.7)		221(40.3)			NA	.90
Feeding times[b]	Times		<8 times	8-12 times		>12 times		<8 times	8-12 times		>12 times			
	Time point	within 28 hours after delivery	84(15.6)	298(55.5)		155(28.9)		88(16.1)	301(54.9)		159(29.0)		NA	.97
		during 28-52 hours after delivery	20(3.7)	328(61.1)		189(35.2)		34(6.2)	336(61.3)		178(32.5)		NA	.14
		during 52-76 hours after delivery	10(1.9)	301(56.1)		226(41.2)		15(2.7)	307(56.0)		226(41.2)		NA	.65
Newborn weight[c], mean (SD), g	Time point	28.5 hours after delivery	3197.304(353.115)					3177.755(360.328)						.37
		52.5 hours after delivery	3150.076(358.292)					3130.942(364.875)						.38
		76.5 hours after delivery	3147.305(360.313)					3126.938(369.281)						.36

[a]Calculated using Fisher exact test.
[b]Calculated using Wilcoxon rank-sum test.
[c]Calculated using *t* test.

© 2022 Zhu Y et al. *JAMA Network Open.*

eTable 2. Subgroup Analysis for Participants With or Without Spinal Analgesia Lactation

**Lactation**																									
Lactation initiation time[a], median (IQR), mins		438.0 (84.00- 1247.00)					367.0 (124.00- 1419.50)					NA	.35	206.5(67.00- 1491.25)					746.0(57.00- 1559.00)					NA	.39
Breastfeeding milk volume[b]																									
Volume		-	+	++	+++	++++	-	+	++	+++	++++			-	+	++	+++	++++	-	+	++	+++	++++		
Time point	28.5 hours	57 (13.0)	289 (66.1)	90 (20.6)	1 (0.2)	0 (0.0)	78 (18.4)	291 (68.8)	49 (11.6)	5 (1.2)	0 (0.0)	NA	<.001	10 (10.0)	72 (72.0)	18 (18.0)	0 (0.0)	0 (0.0)	19 (15.2)	93 (74.4)	12 (9.6)	1 (0.8)	0 (0.0)	NA	.15
	52.5 hours	6 (1.4)	102 (23.3)	263 (60.2)	64 (14.6)	2 (0.5)	9 (2.1)	120 (28.4)	247 (58.4)	46 (10.9)	1 (0.2)	NA	.21	1 (1.0)	13 (13.0)	63 (63.0)	23 (23.0)	0 (0.0)	1 (0.8)	34 (27.2)	75 (60.0)	15 (12.0)	0 (0.0)	NA	.01
	76.5 hours	0 (0.0)	26 (5.9)	132 (30.2)	206 (47.1)	73 (16.7)	1 (0.2)	32 (7.6)	168 (39.7)	186 (44.0)	36 (8.5)	NA	<.001	0 (0.0)	3 (3.0)	24 (24.0)	45 (45.0)	28 (28.0)	0 (0.0)	8 (6.4)	40 (32.0)	67 (53.6)	10 (8.0)	NA	<.001
Feeding mood[b]																									
Mood		Exclusive breastfeeding		Partial breastfeeding			Exclusive breastfeeding		Partial breastfeeding					Exclusive breastfeeding		Partial breastfeeding			Exclusive breastfeeding		Partial breastfeeding				
Time point	within 28 hours after delivery	234(53.5)		203(46.5)			217(51.3)		206(48.7)			NA	.54	55(55.0)		45(45.0)			57(45.6)		68(54.4)			NA	.18
	during 28-52 hours after delivery	208(47.6)		229(52.4)			207(48.9)		216(51.1)			NA	.73	50(50.0)		50(50.0)			54(43.2)		71(56.8)			NA	.35
	during 52-76 hours after delivery	258(59.0)		179(41.0)			260(61.5)		163(38.5)			NA	.49	60(60.0)		40(40.0)			67(53.6)		58(46.4)			NA	.35
Feeding times[b]																									
Times		<8times		8-12times		>12times	<8times		8-12times		>12times			<8times		8-12times		>12times	<8times		8-12times		>12times		
Time point	within 28 hours after delivery	62(14.2)		241(55.1)		134(30.7)	67(15.8)		233(55.1)		123(29.1)	NA	.74	22(22.0)		57(57.0)		21(21.0)	21(16.8)		68(54.4)		36(28.8)	NA	.34
	during 28-52 hours after delivery	15(3.4)		260(59.5)		162(37.1)	24(5.7)		259(61.2)		140(33.1)	NA	.18	5(5.0)		68(68.0)		27(27.0)	10(8.0)		77(61.6)		38(30.4)	NA	.54
	during 52-76 hours after delivery	8(1.8)		234(53.5)		195(44.6)	11(2.6)		233(55.1)		179(42.3)	NA	.64	2(2.0)		67(67.0)		31(31.0)	4(3.2)		74(59.2)		47(37.6)	NA	.50
Newborn weight[c], mean (SD), g																									
Time point	28.5 hours after delivery	3208.128(344.553)					3194.622(360.173)					NA	.57	3150.000(386.624)					3120.680(356.383)					NA	.56
	52.5 hours after delivery	3159.007(345.805)					3145.433(365.589)					NA	.58	3111.050(408.044)					3081.904(359.570)					NA	.58
	76.5 hours after delivery	3155.714(353.283)					3145.288(366.422)					NA	.67	3110.560(389.302)					3064.840(373.621)					NA	.37

[a]Calculated using Wilcoxon rank-sum test.
[b]Calculated using Fisher exact test.
[c]Calculated using *t* test.

© 2022 Zhu Y et al. *JAMA Network Open.*
