# Supplementary material for: Effect of Acupoint Hot Compress on Postpartum Urinary Retention After Vaginal Delivery: A Randomized Clinical Trial
Source: JAMA Netw Open. 2022 May 23;5(5):e2213261. doi: 10.1001/jamanetworkopen.2022.13261 (PMC9127553; doi:10.1001/jamanetworkopen.2022.13261)
Supplement: Supplement 3. — Data Sharing Statement [file jamanetwopen-e2213261-s00.pdf]

Data Sharing Statement

Zhu. Effect of Acupoint Hot Compress on Postpartum Urinary Retention After Vaginal Delivery. *JAMA Netw Open*. Published May 23, 2022. doi:10.1001/jamanetworkopen.2022.13261

Data
Data available: No
